# Further empirical data for torsion on bowed strings

**DOI:** 10.1371/journal.pone.0211217

**Published:** 2019-02-04

**Authors:** Robert Mores

**Affiliations:** Faculty of Design, Media, and Information, Hamburg University of Applied Sciences, Hamburg, Germany; Julius-Maximilians-Universitat Wurzburg, GERMANY

## Abstract

Research on bowed string motion focuses on transverse waves rather than on torsional waves. These are believed to play only a minor role for stabilizing vibrations and no role for perception. Here, torsion is measured on both sides of the bow contact point for a variety of bridge-bow distances on a cello string. Every periodic string release is preceeded by a reverse torsional motion independent from bowing position or dynamics. Transverse and torsional motions are coupled and there are cases of stabilization, but also cases of perturbation or surrender. Structural and timing analyses of torsional waves suggest that the earlier concepts of differential slipping can be essentially confirmed while the concept of Schelleng ripples cannot be confirmed and the concept of subharmonics is under question.

## Introduction

In bowed string motion not only transverse waves but also torsional waves are excited due to the tangentially applied force at the surface of the string. Early observations of torsion date back more than 100 years as reported by Cremer, chap. 6.1 in [1]. String torsion is generally believed to have little impact on sound, because a rotating string itself will not radiate. The body of the instrument will not radiate either, because the moment to the bridge as determined by the string diameter is very small [2]. Torsion is nevertheless believed to have a stabilizing effect on periodic bowed-string motion [3]. Torsion has therefore played a role in models and simulations of bowed strings. The most recent discussion concludes in the need for more elaborate friction models that could possibly be combined with the already investigated effects of a limited bow width [4]. While these models gain in fidelity, this study seeks to draw some conclusions from experiments and to revisit the relation of general observations and existing concepts. Among these concepts, which relate to torsion, are bow force limits, Schelleng ripples, subharmonics and Friedlander’s instability, limited bow hair width and differential slipping, and friction models.

Bow force limits refer to the minimum and maximum bow forces that would allow to maintain Helmholtz motion for a particular bow velocity *v*_*b*_ and bowing position *ß* relative to the length *l* of the vibrating string. The translational impedance *Z*_*0*_ of the string is one of the key parameters in the model, apart from the termination impedance *R* and the friction coefficients. Schoonderwaldt extended existing models based on the argument that the true tangential impedance *Z*_*tot*_ at the contact point should be somewhat lower than the translational impedance since the string is willing to rotate sidewards when the tangential bow force applies, *Z*_*tot*_ = *Z*_*0*_*Z*_*R*_/(*Z*_*o*_+*Z*_*R*_), with *Z*_*R*_ the torsional wave impedance [5]. This model is still used today in enhanced wave-based bowed-string modelling [6]. Recent, more precise measurements suggest that this extension cannot be confirmed at least when it comes to predicting the maximum bow force [7]. The argument is rational for a quasi-static model but obviously does not hold for the true stick-slip interaction given the dynamics of transverse and rotational waves travelling along the string.

**Fig 1:**
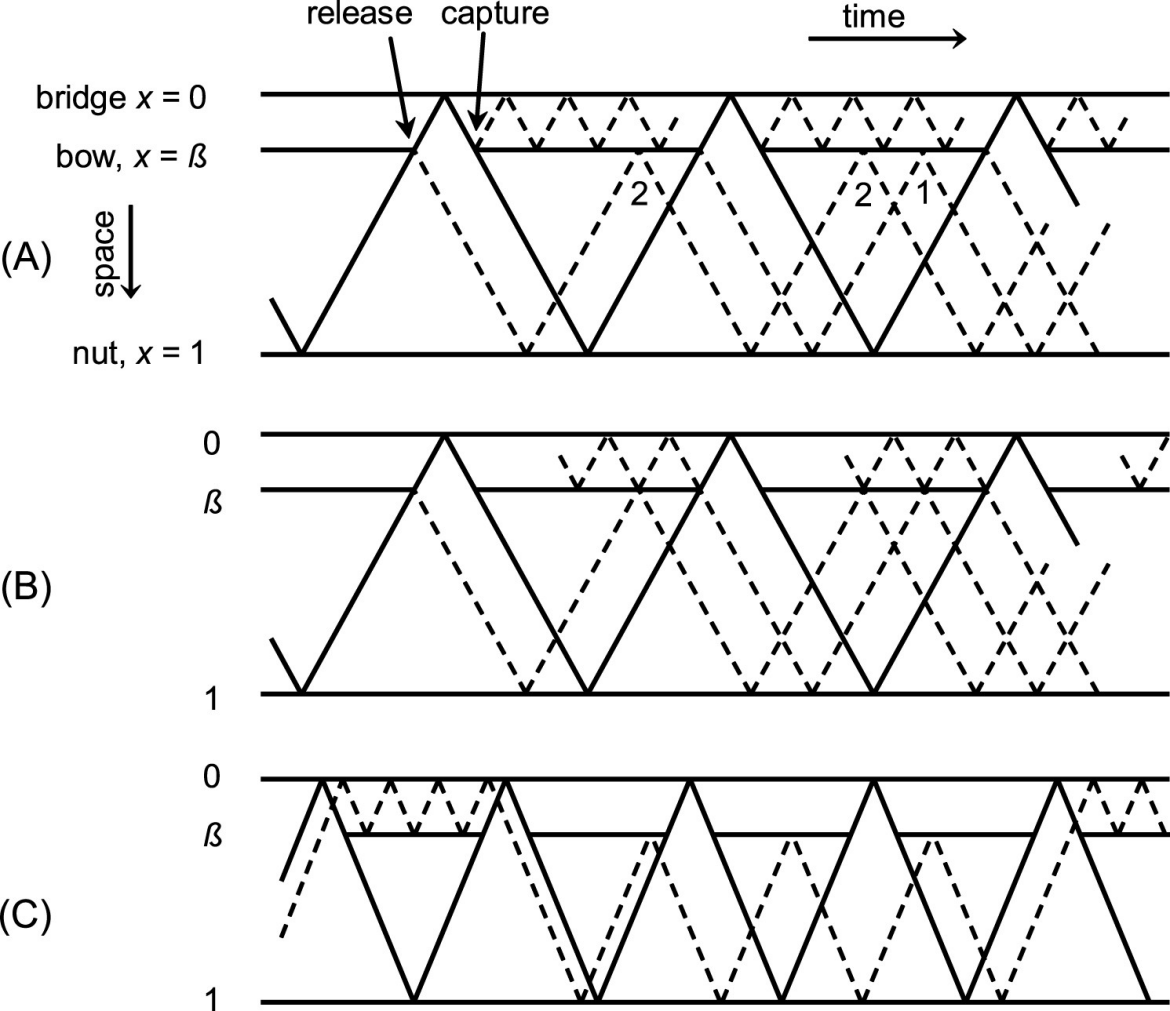
Path of a Helmholtz corner in the space-time diagram. Heavy lines at bridge and nut indicate reflecting barriers, likewise the bow at position *ß* in times of sticking. Paths of primary (―) and secondary (---) waves. (A) Principle of declining bridge-sided reflections at the beat of the recent stick-slip action and principle of nut-sided younger (1) versus older (2) Schelleng ripples. (B) Observation in this study with bridge-sided reflections in phase with upcoming releases. (C) Principle of subharmonics, secondary waves that trespass underneath the bow in times of slipping. (A) and (C) reproduced from [9].

The concept of the Schelleng ripples follows the argument that a wave arriving from the nut cannot because of its rounded corner immediately release the string from the bow [8]. During the finite time it takes to build up the necessary threshold force the wave is reflected. Reflections from the bow travel to the nut and return again to the bow, arriving there just before the next release. In his bridge force recordings Schelleng observed that the ripple grows in the vicinity of an upcoming release. His explanation is that the individual ripple last to the instant of release is the one arising from the most recent wave reflection while the last but one ripple is the one arising from the last but one Helmholtz cycle, and so on. The last but one ripple experienced one additional cycle of reflections and associated damping and therefore has a smaller amplitude. The ripple diminishes over time, and only the temporal sequence of their appearance evokes the impression of a growing ripple. The time scheme of such occurrences is illustrated in Fig 1A, see Fig 8 in [9]. The youngest ripple relative to an upcoming release is denoted with the number 1 and the second youngest ripple is denoted with the number 2 in Fig 1A.

Fig 1B represents findings of this paper and is shown at this early point only to facilitate the direct comparison with Fig 1A.

The underlying assumption of this concept is that waves have a chance to travel and to be reflected several times over the course of several main cycles, without being disturbed by other vibrational modes and without suffering from transformation into other vibrational modes. The argument that the bow is in contact with a string’s surface rather than with its center is widely accepted for modelling simultaneous excitation of transverse and torsional waves, and the same argument also holds for the transformation of one mode of vibration into another.

Subharmonics are audible deviations from strict periodic cycles, for instance deviations that would repeat every four or five cycles. The concept of subharmonics can be explained with the schematic of reflected waves, too, see Fig 1C, following Fig 11 in [9]. Waves arriving at the bow during sticking are likely to be reflected while waves arriving during slipping are likely to be transmitted across the contact area. The windows of opportunity for such transmission relate to *ß* and determine the geometry of subharmonic paths. Woodhouse argues that Friedlander’s instability of Helmholtz motion [10] can be explained by slight amplifications of incident disturbances. Such amplification rises from a negative resistance [8] which can be explained by the nature of the friction curves. Even though developed several decades ago, the concepts of Schelleng ripples and subharmonics are still the basis in recent enhanced simulations [6]. Again, this concept only works if transverse waves are travelling and are reflected several times over the course of several main cycles without being disturbed by other vibrational modes and without suffering from transformation into other vibrational modes.

Guettler [11] used the numerical simulation of McIntyre et al., [9], [12], and [13], to investigate mechanisms of subharmonics and cycle prolongation caused by torsion, on the basis of two strings of sligthly differing properties. He sketches cases of mutual transformations between transverse and torsional pulses, and cases of anomalous low frequencies triggered by coinciding transverse and torsional impulses in cases of high bow pressure. He further studied the parameters for perfect onsets by simulation in [14], remarking the difficulty to appropriately model the torsional component while using the “parallel reactance” model for *Z*_*o*_ || *Z*_*R*_. As mentioned above this model failed to predict maximum bow force.

Tracing further back, Cremer expected that all “secondary” transverse and torsional waves can be reflected and mutually transformed at the ends of the string and the contact point, see chap. 6.7 of [1]. The resulting space-time diagram is densly populated with secondary waves even for the drawn sparse case of exactly matching integer multiples for the relation of torsional to transverse wave velocities. He expects space-time diagrams of any desired density for irrational velocity relations. Nevertheless, Cremer assumed the possibility of multiple reflections of secondary waves within a main cycle but also across several main cycles which implies assumptions of only litte damping. Furthermore, he draws a picture of sparser secondary waves populations for cases of absorption of torsional waves at string terminations, which implies the assumption that torsional waves are less likely to survive multiple reflections as compared to transverse waves.

The concept of limited bow width and the consequent differential slipping is an important element in stick-slip modelling. Pitteroff and Woodhouse [15] modelled the contact area across the bow width to improve earlier models of a single point of contact. The given width of a bundle of bow hairs in contact with the string suggests that there is no such concerted stick-slip action as ordinarily anticipated since the string, in conjunction with its gradual movement during sticking, also gradually changes its angle relative to the bow. The geometry of string movement therefore implies a displacement and a quasi-static force, see Fig 3 in [15]. Assuming a single point of contact within the given width of the hair bundle, this force is directed towards opposite directions at the inner and outer edge of the bundle, resulting in differential slipping, directed backwards and forwards relative to bow motion, respectively.

**Fig 2:**
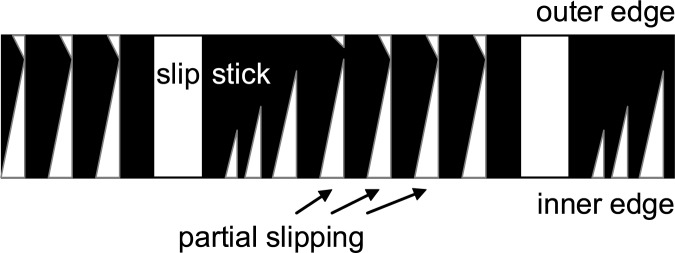
Multiple differential slipping in the course of fundamental stick-slip cycles. Essential results extracted from simulations on limited bow width [15], indicating that differential slipping occurs at the inner edge, on the bridge side.

The bowing simulation based on the finite width model also incorporates flexible and stiff strings. Simulations also build on the fact that transverse and rotational waves depart from the contact into two directions and that these are not only reflected at the bridge and the nut, but also again at the bow while they return. This reflection and transmission behaviour at the contact point is carefully modelled, as well [16]. The schematic in Fig 2 qualitatively reproduces the main results of simulations across variations of bow force and bridge distance as can be found in [15]. As illustrated, within each complete Helmholtz cycle, i.e. an alternating slip and a stick across the entire width of the bow, there are interim slips along parts of the contact area. The action seems to be stronger at the inner edge of the bow hair and seems to be synchronized at the inner and outer edge which is reasonable given the bending stiffness of the string.

Friction models play an increasingly important part in the simulation of bowed strings. In contrast to earlier models of a hyperbolic relation between friction coefficient and sliding velocity, Smith and Woodhouse proposed an empirically fitted function and, seeking higher fidelity of simulated bridge force, also an alternative thermal friction model [17]. The thermal model assumes that the friction force is governed by a plastic yield process, with a yield strength that is a function of contact temperature. Mansour’s recent simulations conclude that the choice of the friction model is by far the most sensitive parameter to bow force limits, pitch flattening and relative spectral centroid [6]. No other parameter such as allowing or not allowing torsion, bending stiffness, or rigid termination did change the simulation outcome as much as did the friction model. Apart from seeking the friction models of higher fidelity the problem of combing enhanced friction models together with the approach of limited bow width seems to be challenging. Mansour’s simulation used the single-point contact model, giving up some of the achievements of the limited bow width model. This dominating importance of a suitable friction model is also claimed in another recent study where alternative friction models are investigated in how well these predict stick-slip action in the context of the Schelleng diagram, considering bow force limits and vibrational modes, and in the context of the Guettler diagram, considering the spontaneity of establishing Helmholtz motion [4]. The authors conclude in further developing improved friction models. They also conclude that ‘one must fall back on assessing the candidate models based on empirical evidence, since it must be accepted that no current model has a complete and secure basis in physics’.

This study seeks more empirical evidence while measuring string excitation and torsion across some parameters of bowing. Most of the earlier studies preferred bridge force measurements to derive conclusions. Here, excitation is measured directly at the contact point, and the torsion is measured in the vicinity of the contact point. The idea is not new but it will be applied in a way to capture torsion on both sides of the contact point in order to analyze possible causes of action. Woodhouse et al. have shown that action at the bowing point can be traced back from bridge force measurements [18], so such analysis should be possible with the existing simulation models as well. This study trusts that temporal issues can be better observed and explored at the point of origin, at the contact point, rather than remotely. It seeks empirical findings to understand a little bit more of the behaviour of transverse and torsional waves in the typical playing range in the light of the existing concepts.

After some brief statements on method, measurement and instrumentation issues, one discussed data for the conventional playing range, and another section for playing outside the conventional range. A final section gives a few suggestions for future modelling before concluding.

## Method and instrumentation

For most measurements a cello G steel string is used, mounted to a monochord and bowed by a real bow. The monochord is preferred over a real cello because incorporated body resonances would otherwise increase the complexity of vibrational modes and aggravate the interpretation of impulse patterns and stick-slip interactions. String excitation is measured underneath the contact point and string torsion is measured in the vicinity of the contact point, both via electric pick-ups. This location is preferred as to picture impulse patterns departing from and returning to the contact point.

Measurements are then reasoned against the velocity properties of the string and impulses are classified, before drawing first conclusions. Further specific experiments are conducted to confirm findings where necessary.

### Instrumentation setup

The string under investigation is mounted on a monochord made of solid steel. The weight of the monochord is approximately 2 kg. A brief check with an additional mass load of 1 kg at each end of the string did not show any significant difference in terms of sustain or harmonic sound structure concluding that the mass and stiffness boundary conditions imposed by the monochord are good enough for this investigation. The monochord rests on a table with an intermediate layer of leather to dampen peripheral vibrations, if any.

The string termination support accomodates alternative termination materials with different resistances. These have been measured and used in another study [19]. The material used here is a 4 mm thin layer of felt with a steel pin to support the string. The measured resistance, just for guidance and not relevant in subsequent analysis, is *R* = 939±13 kg/s as measured at low frequencies.

### String properties

The cello G string made of steel is a Pirastro Chromcor medium string with properties according to Table 1, most of which are measured.

**Table 1:** Properties of the Pirastro Chromcor Medium cello G steel string when tuned to 98 Hz at *l* = 680 mm.

property	unit	
mass per unit length	g/m	6.15
diameter	mm	1.19
nominal tension	N	121
transverse wave speed *v*_*tra*_	m/s	133
transverse wave impedance *Z*_*0*_	kg/s	0.93
torsional fundamental frequency	Hz	543
torsional wave speed *v*_*tor*_	m/s	738

In experiments, the string is tuned to the nominal 98.0±0.1 Hz at length *l* = 0.68 m. The torsional wave speed did not change notably when varying the tuning in the range of 88 Hz to 108 Hz. Such independence from tension is in accordance with other findings [2]. Damping or Q factors are not measured for the used string. For the purpose of this investigation, however, it is good to recall that torsional Q factors are roughly an order of magnitude smaller than those of the transverse modes of the same string [2]. Therefore, torsional waves are likely to be dampened much stronger than transverse waves.

### Signal measurement

**Fig 3:**
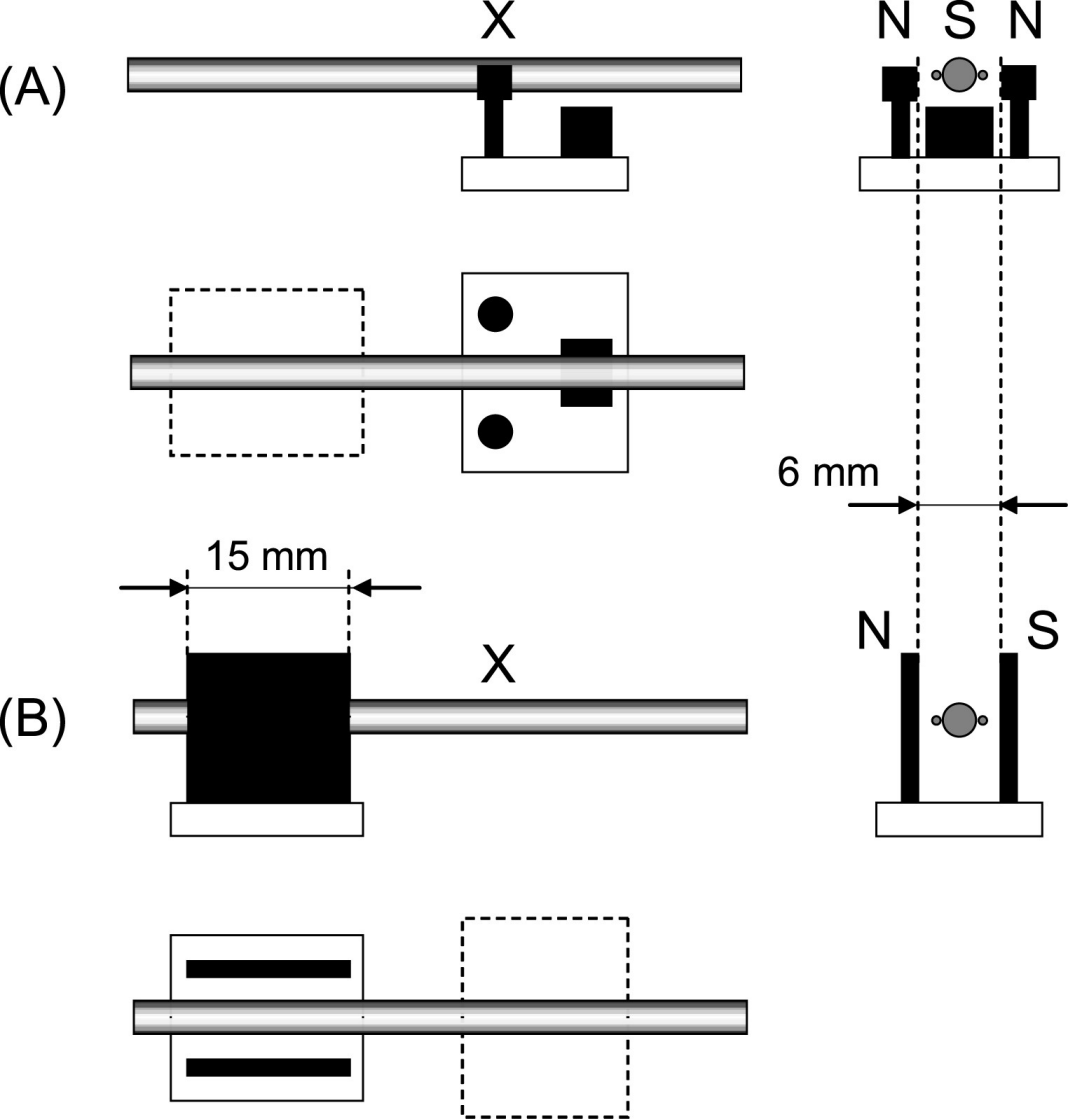
Schematic top and side views of employed electrodynamic pick-ups. (N) north and (S) south poles of permanent magnets, (X) bowing position. (A) Lateral string velocity using Gibson’s patent US 6392137 [20]. (B) Torsional angular velocity using a wire loop attached to the string in a homogenous magnetic field [21]. Scheme not to scale.

String velocity and torsional angular velocity are captured by electric coils which are arranged as indicated by Fig 3. A pickup according to a Gibson patent [20] is located directly underneath the bow contact point and can be moved along the monochord for varying *ß*. This pickup captures string velocity in bowing direction. Other than conventional single coil or Humbucker guitar pickups this pickup captures velocity quite independently from the actual string position. This is achieved, according to the patent, by a smart geometrical arrangement of the direction of the magnetic field in relation to the string and differential winding on a twin pole shoe, Fig 3A. The magnetic flux density between the twin pole shoes and the string is about 11 mT.

Torsional angular velocity is captured by a fine wire loop glued laterally to a few centimeters of the string, up one side and back down the other [21]. Placed in a uniform magnetic field (30 mT), oriented in the plane of the loop, a voltage will be generated. This will be proportional to the differential velocity on the two sides of the string and therefore translate to angular velocity, Fig 3B. Proportionality holds for small amplitudes of motion. The limited width of the pole shoes, *w* = 15 mm, represent a spacial filtering effect, which has been measured separately by a 2 mm short loop and with a continuous torsional rotation, and alternatively by measuring the magnetic flux density with industry standard instrumentation. For homogeneity, 3 dB and 10 dB widths of this spacial filter, see Table 2.

**Table 2:** Spacial properties of torsional pickup with 15 mm wide pole-shoes.

	homogeneity across 10 mm span	3 dB width	10 dB width
measured by a loop on rotating string, loop length ≈ 2 mm	+0 / -0.4 dB	15.5 mm	21 mm
measured magnetic flux density B, sensor width < 5 mm	+0 / -0.4 dB	16 mm	23 mm

The copper wire has a diameter of 50 μm and its total weight is 2 mg for the mounted length of two times 0.18 m. Estimating the same weight for the flexible thin layer of glue, the total mass load on the string is 4 mg. This corresponds to 22 mg per meter, or 0.36% of the string’s mass per unit length, a contribution considered to be small enough to facilitate credible measurements. The position of magnetic pole shoes can be varied with *ß*, and is close to the bowing point on either side, towards the nut or towards the bridge. The distance to the bowing point is preferably 1 cm to sufficiently suppress cross talk between the pickups.

Both velocity signals are integrated with a high-quality charging amplifier with linear characteristics between 3 Hz and 40 kHz (TSC3 from AER, Recklinghausen, Germany), effectively translating the signals to string displacement and torsional angle. These signals are admittedly not linear, however, their relation to the true displacement and the true angle is bijective, a one-to-one relation. This is considered to be sufficient for observing principles of action. Final normalization has been done by displacement measurement and by a torsional step function with predefined angle. The results are only roughly estimated to allow readers to sense the magnitude of motion.

More important, the extent of crosstalk has been controlled. This is important because results should be unequivocally interpreted to result from torsion or from transverse motion either. The crosstalk from torsion to displacement is -25 dB, i.e. a pure torsional motion without displacement will still elicit a displacement signal, however 25 dB below the level of the torsion signal. The crosstalk in the other direction is -15 dB. For instance, ±0.5 mm mechanical displacement (40Hz) delivers 0dB for the displacement signal and not more than -15dB for the torsion signal, and, the other way around, ±4° mechanical torsion delivers 0dB for the torsion signal and not more than -25dB for the displacement signal. All measurements are done in situ with the same string and wire loop, while using an exciter rather than a bow.

## Data for the regular playing range

### Raw data and parameters of bowing

**Fig 4:**
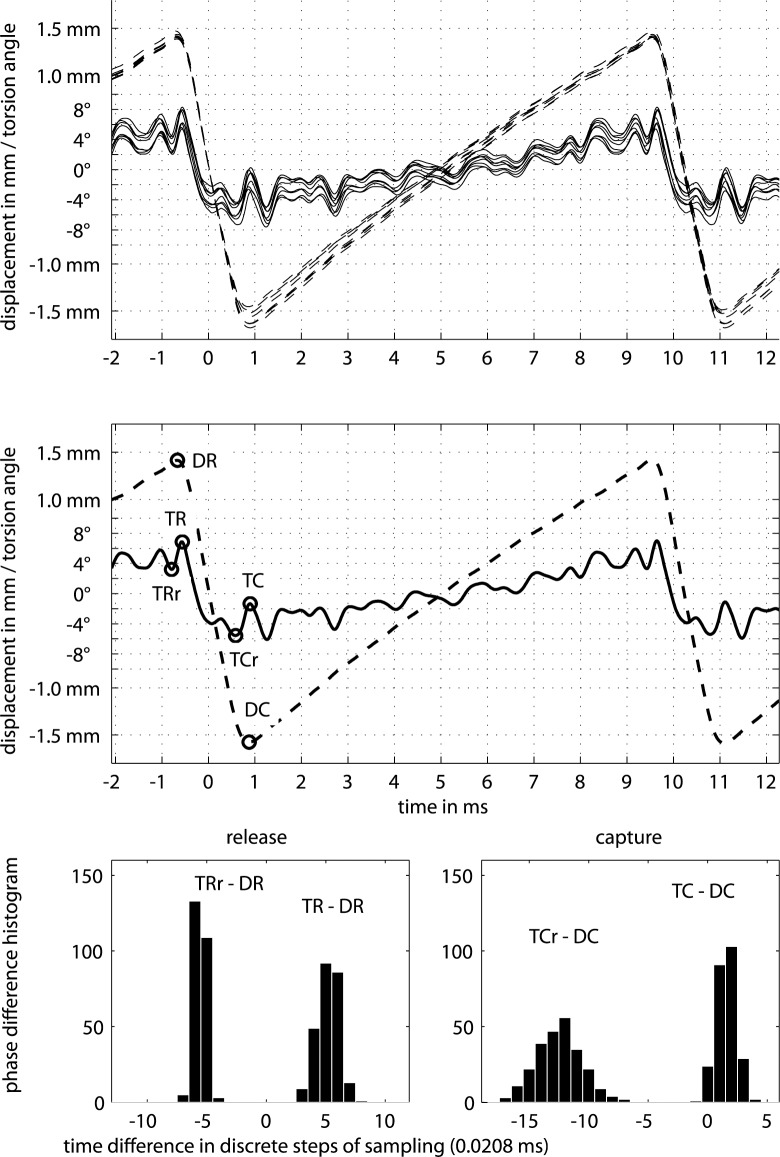
String displacement, torsion and phase relations between them. String displacement (---), electrically measured at the contact point, and torsion angle (―), measured 10 mm away from the contact point towards the bridge side, for an open cello G steel string (*f*_*0*_ = 98 Hz, *T*_*0*_ = 10.2 ms) when bowed at *ß* = 80 mm with an upstroke, showing eight consecutive cycles (top) and the average of these eight cycles (center), with peaks of interest: release (DR) and capture (DC) of the string in the displacement signal, torsion during release (TR) and reverse torsion during release (TRr), as well as torsion during capture (TC) and reverse torsion during capture (TCr) in the torsion signal. Related histograms of the time differences between peaks of interest during release (bottom left) and capture (bottom right) across some 250 cycles.

A typical recording of an upstroke on the open G string is shown in Fig 4. Zero on the time axis relates to the zero crossing of string displacement during slipping. Positive displacements and angles are directed towards bowing direction. Note that the zero crossing does not necessarily refer to the string in rest position since the signal chain involves an integrator. The string might even not reach its rest position, especially when bow forces are high. So the zero crossing referes to the mean of an AC-coupled signal. The upper graph shows an overlay of eight consecutive periods, each triggered at its individual zero crossing. The center graph shows the typical representation of torsion and displacement by simply averaging such eight periods. The bottom graphs indicate the statistics of phase relations between torsion and displacement across some 250 cycles.

There are a few first observations. Torsion seems to follow the general trend line of the periodic sawtooth. This means that the displacement and the associated force applied to the string also causes quasi-static torsion. A brief experiment confirms this observation for the quasi-static case: with a bow resting on the string and slow manual back-forth motion (no bow movement relative to the string, i.e. no slipping), the two signals of torsion and displacement are monitored to be identical, apart from normalization. This observation is in accordance with what can be expected for the string when its tension grows with displacement. The question raises whether this is true also for the dynamic stick-slip action.

Referring to the simulations of [18], the question of how much of a quasi-static cyclic ramp is contained in the torsional signal is not resolved. Their simulation concludes in a relative displacement between rod and string during the sticking phase which reveals a steady movement of the string while sticking to the driving rod, in their Fig 8B. The string steadily falls back in the same direction as slipping, which can be well explained with a string that is gradually rolling during sticking. But it can also be explained by a string that hops backwards without torsion.

**Fig 5:**
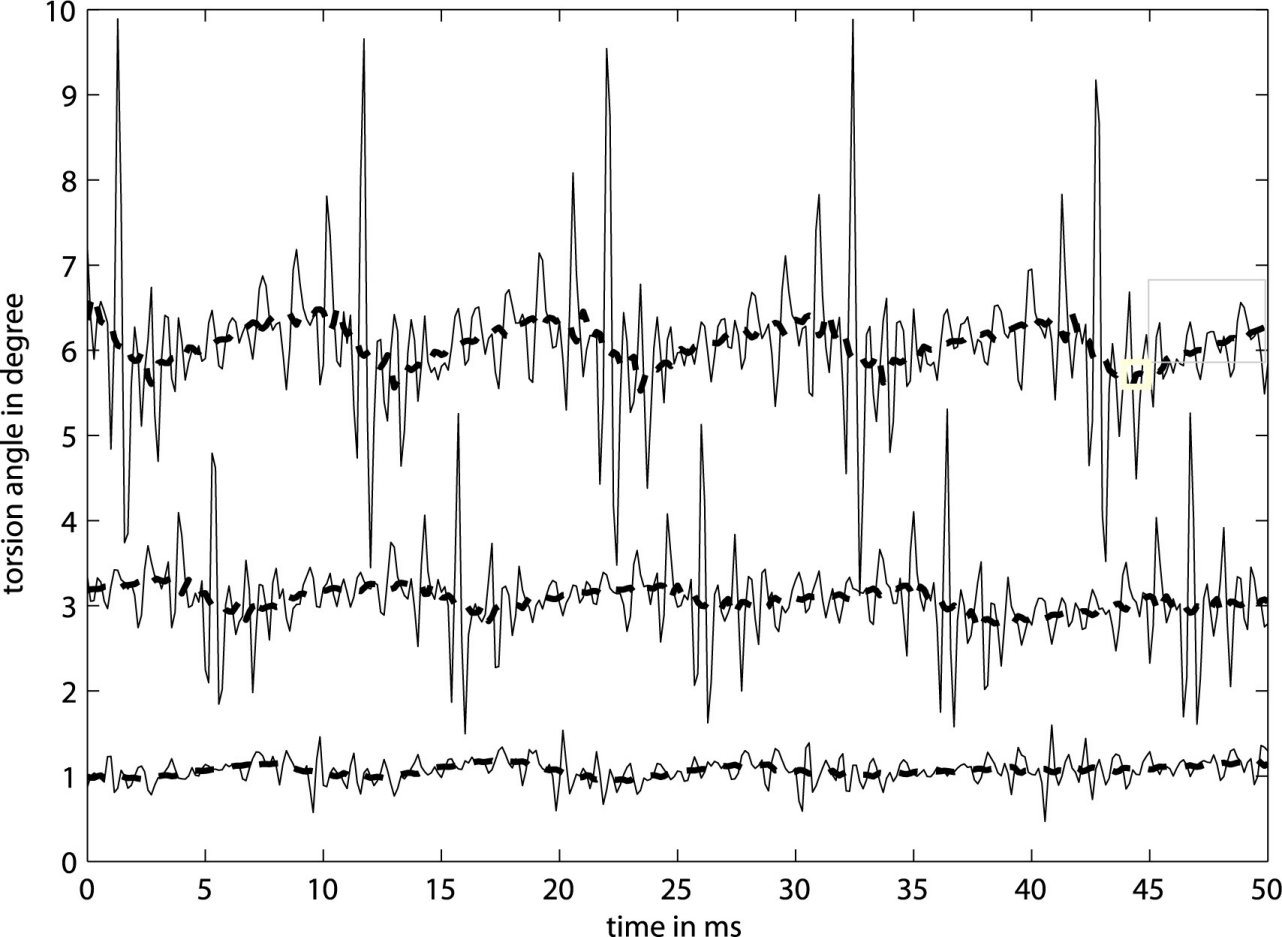
Torsion on an open cello G string measured optically. Torsion (―) on an open cello G steel string when bowed manually at *ß* = 80 mm at three different levels of bow force and associated bow velocity, as measured by a high speed camera at 7000 frames per second at d = 70 mm from the bridge. The same signals with a moving average filter of width 15 (---). Traces from the bottom to the top refer to low, medium, and high bow force.

To resolve this question, additional high-speed videos were taken at 7000 frames per second for the same string and the same parameters as used in Fig 4. Fig 5 shows the torsion angle for manual bowing at three different levels of bow force. Raw data and extracted motion tracks are hosted under [22]. Displacement and ripples can be confirmed, and also the cyclic ramp is given, however not to the extend as indicated by Fig 4. While peak-to-peak ranges are comparable for the torsional vibration, the peak-to-peak ranges for the ramp are only about 1° when measured optically versus roughly 8° when measured electrically. A good portion of the ramp in Fig 4 is therefore assumed to come from crosstalk, which can be explained by the crosstalk outlined above.

The other apparent observation is that there is no regular torsional wave but there are short sections of harmonic torsional oscillation just before and after the slipping while the rest of the trace seems to be non-harmonic. The overlay of consecutive periods reveals the deterministic character in the non-harmonic segments, if the reader is willing to ignore the minor inter-trace DC-shifts resulting from instrumentation. The character of the torsion signal depends strongly on *ß* but does not vary much with bow force, or with bow velocity. This observation of determination is true for the entire range of *ß* in the typical playing range.

Another observation is that the amplitude of torsional oscillation and therefore the associated ripple in the displacement trace grows while the release comes nearer. This can be observed in bridge force measurements of other investigations as well, see Fig 2 in [8], and [9]. Whether such growing results from the concept of younger vs. older Schelleng ripples, or from differential slipping, see Figs 13 and 15 in [15], will be discussed later.

In the graph, points of return for displacement and torsion are indicated for further discussion. For example, DR marks the point of return regarding the displacement during release action, and DC marks this for the capture action. TR corresponds to the point of return of torsion during the release action, TC for the capture action. Both maxima represent torsional angles the direction of which agrees with the current bowing direction. TRr and TCr represent minima that directly precede these maxima with a direction reverse to bowing which might indicate slipping. Phase histograms indicate the time difference between maxima of torsion and displacement for 250 consecutive cycles, with bins given by the sampling rate of 48 kHz. Visual inspection suggests normal distribution which is confirmed by a chi-square goodness-of-fit test at 5% significance. As can be seen in the graph, the instances of DR and TR are closely related as well as DC and TC. This will later be explored more systematically across the range of *ß*. Nevertheless, it can already be concluded that actions of torsion and displacement are deterministically related.

**Fig 6:**
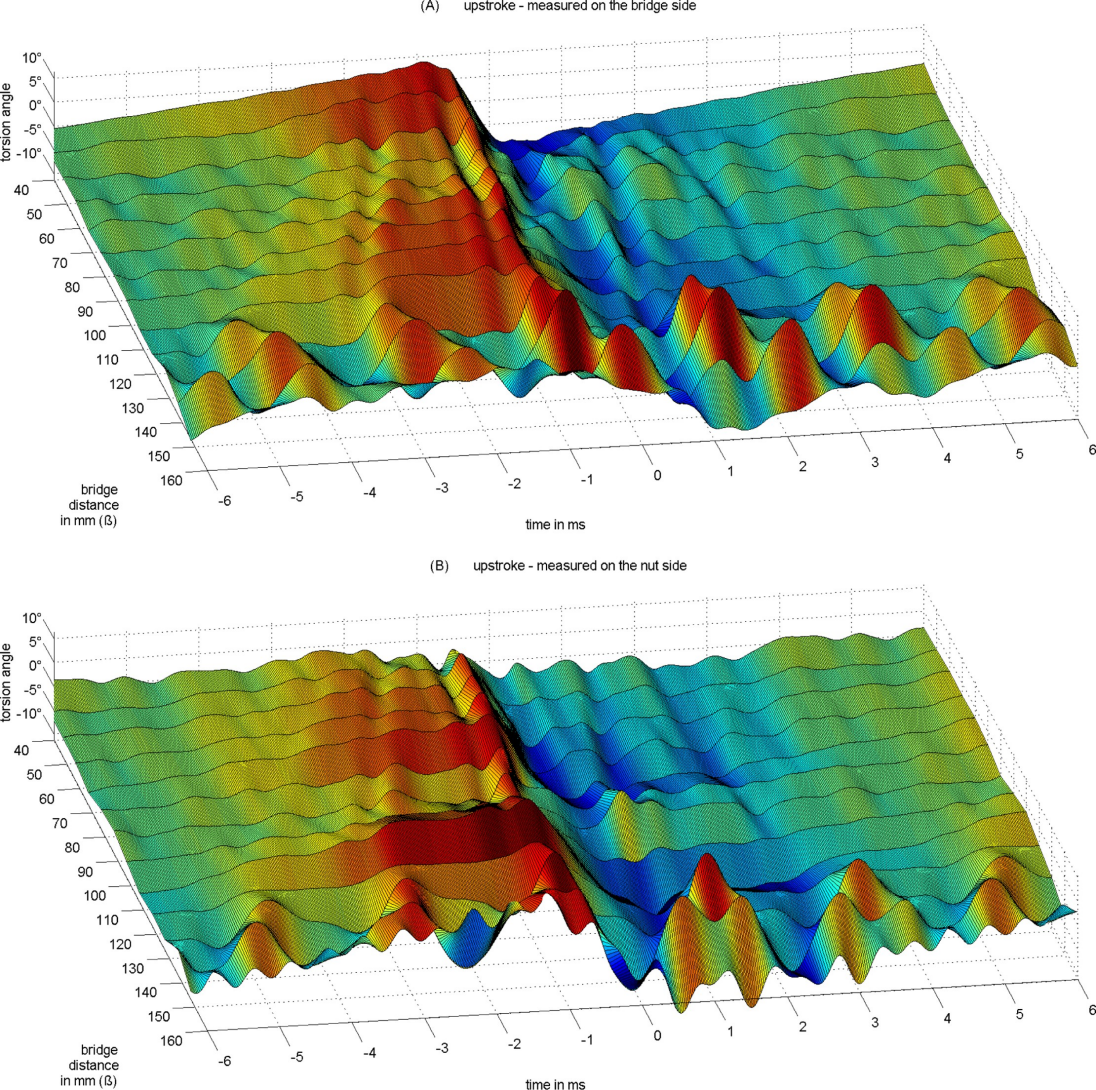
Torsional vibration of an upstroked open cello G string (*f*_*0*_ = 98 Hz) on a monochord for varying *ß*. Pickup position 10 mm away from the contact point towards (A) the bridge and towards (B) the nut. Reference at 0 ms is the zero-crossing of the respective displacement signal during release (not shown here).

**Fig 7:**
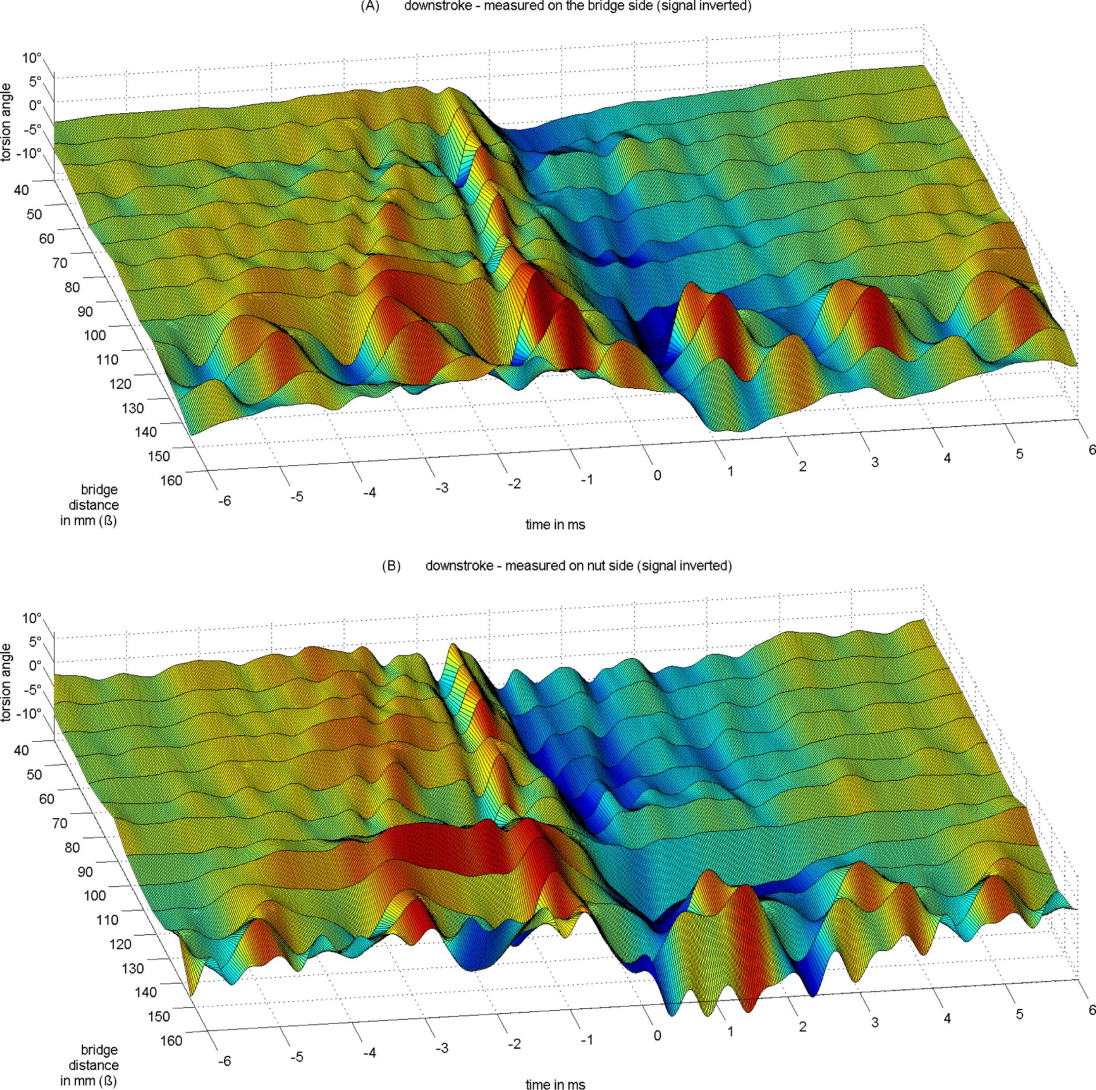
Torsional vibration of an downstroked open cello G string (*f*_*0*_ = 98 Hz) on a monochord for varying *ß*. Pickup position 10 mm away from the contact point towards (A) the bridge and towards (B) the nut. Reference at 0 ms is the zero-crossing of the respective displacement signal during release (not shown here). Downstroke signals are inverted for ease of comparison with graphs in Fig 6.

The torsion trace in Fig 4 reveals a structural organization which can be visually inspected when plotting the torsion for Helmholtz cycles across the spread of *ß* from 40 to 160 mm relative to 680mm string length, see Figs 6 and 7, [23] The individual trace from Fig 4, an upstroke at *ß* = 80 mm measured at the bridge side, is now just one among many in Fig 6A. Each trace results from the same averaging across eight consecutive cycles, without any normalization. The total of 52 individual manual down- and upstrokes comes with varying bowing velocity and bow force. Figs 6 and 7 show torsion traces taken from sections of the set of up- and downstrokes where displacement amplitudes have a comparable level.

While reasoning the contextual structural organization of torsion, up- and downstrokes are considered, as well as measurements on the bridge side versus measurements on the nut side, Figs 6 and 7.

By visual inspection, there are similarities but also differences between up- and downstrokes, and between bridge side and nut side. For the discussion here, the focus is on reasoning earlier concepts. The structure seems to follow rules in relation with *ß* in the range from 40 mm to 120 mm, the typical playing range, which will be explored in the next sub-section. Within this range, the structure appears to be more or less regular apart from the cycle at *ß* = 100 mm where the torsional wave is somewhat deteriorated, see Fig 7B, the upstroke measured at the nut side. The reason for this will be explored in a following sub-section. A later sub-section reasons the cause of action for release. The question is which of the transverse or torsional impulses on either side of the contact point is likely to trigger actions of slipping or differential slipping. The phase relations are believed to answer some of the questions. Another sub-section briefly discusses phase relations during capture. The structure and the phase relations both seem to systematically change at *ß* = 130 mm and above. This comes from driving the bow at approximately *ß* = 1/5 = 136 mm. A final sub-section investigates potentially existing subharmonics.

### Structural organization of torsional waves

The visual inspection of Figs 6 and 7 suggests that the timing of individual torsional impulses relates to *ß*. Before starting interpretations there is a necessary preliminary note to be made. The measured torsion signal strictly records torsion, as mentioned above, with only -20dB crosstalk. However, transverse waves are very well visible too because any transverse motion in the vicinity of the contact point during sticking will directly translate to torsion, which is then measured. Therefore, the torsion signal reveals both types of motion.

**Fig 8:**
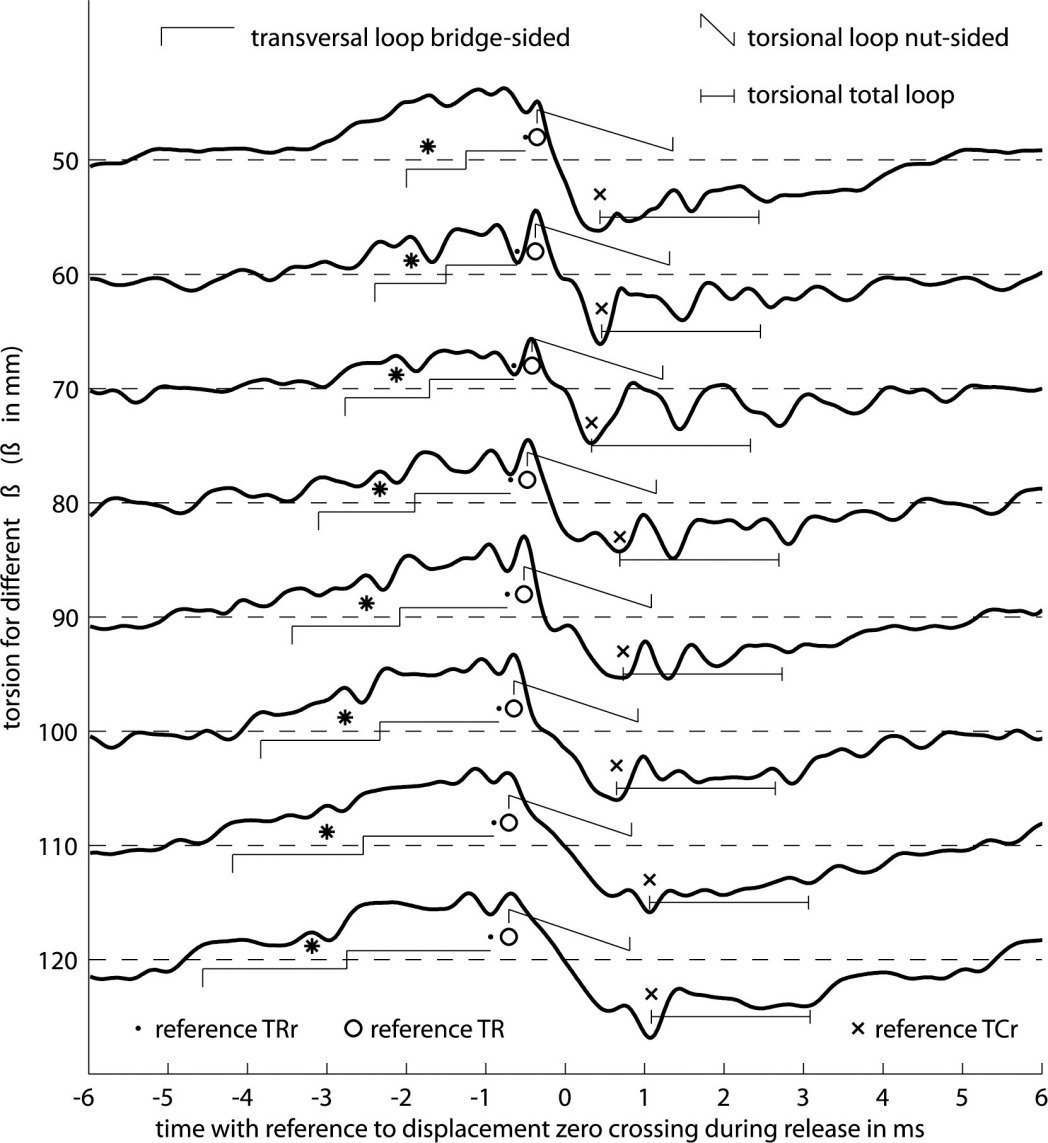
Temporal analysis for torsional vibration on the open cello G string. Torsional vibration for varying *ß* measured during upstrokes 10 mm away from the contact point towards the bridge (corresponds to Fig 6A). The graph includes symbolic bars for diverse loop times (see legend) to support the visual inspection of the impulse pattern and its underlying structure. References (**∙**), (**○**), and (**×**) correspond to Fig 4. Reference (*) for discussion in the sub-section ‘local perturbation’.

Starting with the inspection, there are two major steps prior to release. The width of these grows with *ß*. Mapping known travelling- and loop-times for torsional and transverse waves to the measured signals leads to Fig 8. From the measured velocities for the torsional and transverse waves the respective travelling- and loop-times are derived for varying *ß*, see Table 3. Loop-times are 10.2 ms for the transverse, 1.9 ms for the torsional and estimated 0.5 ms for the longitudinal waves.

**Table 3:** Loop times of transverse and torsional waves on the open cello G string in relation to *ß*.

*ß* in mm	torsional wave loop time in ms between bow and		transverse wave loop time in ms between bow and	
	bridge	nut	bridge	nut
40	0.11	1.73	0.60	9.62
60	0.16	1.68	0.90	9.32
80	0.22	1.63	1.20	9.02
100	0.27	**1.57**	**1.50**	8.72
120	0.33	1.52	1.81	8.42

Refer to text for numbers printed in bold.

While the interpretation of individual traces might turn out ambiguously, the holistic perspective allows a rather clear assignment of transverse versus torsional modes of vibrations, as well as nut- versus bridge-sided reflections.

For instance, the above mentioned two to three steps prior to slipping correspond well to the loop-times of transverse waves on the bridge side. Two of these loop-times are indicated with reference to TR, the turning point of the torsional motion during the release action. The steps are well represented in the trace of the torsion signal, and the temporal variation that comes from variation of *ß* corresponds well. The steps correspond to a Helmholtz motion for the fraction of the string determined by *ß*. Pitteroff and Woodhouse [15] call this miniature Helmholtz motion (*m-HM*), and they conclude from simulation that differential slipping on the bridge side plays a role in this.

Likewise clearly assignable is the torsional wave that is reflected from the nut side. Again, with reference to TR, the strong torsional impulse returns from the nut side with the same polarity exactly after the measured loop-time. This loop-time diminishes slightly with growing *ß*, and the impulse is on time for all *ß*.

Another clear assignment is that of the torsional wave for the total loop. Very apparent in the graphs of Figs 6 and 7, there is a post-release response pattern which appears to be independent from *ß*. The timing suits the loop-time of torsional waves for the total loop. With reference to TCR, the reverse-to-bowing torsional turning point during capture, the expected impulse returns after the measured loop-time with the expected polarity, for all *ß*. Note that this observation requires the torsional wave to pass the contact point after being reflected on the bridge side, and in fact, two travelling torsional waves work together: one travelling towards and being reflected from the bridge, passing the bow, and being reflected from the nut, and another one travelling towards and being reflected from the nut, passing the bow, and being reflected from the bridge. The reader might identify a second total loop in the graphs of Figs 6 and 7, however the strength diminishes due to damping. However, this is the only oservation of secondary waves being reflected more than once, contrary to the assumptions of earlier studies.

Contrary to this expectation of damping, the impulses prior to release gain in strength. Each reflection of transverse waves, or, each *m-HM* cycle, is accompanied by torsional motion. The torsional motion is identifiable by densely following positive and negative impulses. For instance, the temporal separation of TR and its preceding TRr (not indicated in Fig 8) corresponds well to the loop-time for torsional waves on the bridge side, Table 3. Inspecting Figs 6, 7 and 8, torsional and transverse vibrations seem to work together. Each reflection of transverse waves is accompanied by a preceding stronger-than-usual reverse torsional motion. This reverse torsional motion corresponds to differential slipping. There seem to be several occations of minor differential slipping within each *m-HM* cycle, depending on *ß*, without much further effect, while the stronger-than-usual reverse torsional motion is synchronous with the *m-HM* cycle. The stronger-than-usual reverse torsional motion can be explained by the reflected transverse wave that is just about to arrive at the contact point and which works in the same direction. Remember that a transverse motion in bowing direction will cause a backward rotation due to the vicinity of a contact point. In other words, the differential slipping is stronger in times when the reflected transverse wave is about to return to the bow. This can be observed in most graphs and for almost every *ß*. The torsional wave, in return, co-determines the *m-HM* cycle. Now, while the cycle will take place with or without torsion, a good portion of what is identified as the slope of a *m-HM* cycle is accredited to torsion. So at least the micro-timing of the cycle is determined by torsion. Similar but not the same findings on mutual synchronisation were found by Pitteroff and Woodhouse, p. 756 in [15].

The same observation of mutual co-determination also holds for the fundamental Helmholtz cycle. The last reverse torsional impulse prior to release is the strongest. The approaching transverse wave, which is reflected from the nut and which determines the main cycle, also works in the same direction of an increased differential slipping. This differential slipping is again co-determining the micro-timing. It also co-determines corner rounding of the sawtooth which until now has only been discussed and modelled on the basis of transverse waves.

Many other impulses cannot be explained that easily, because of diverse origin and, at the same time, instantaneous transformation. Remember that each transverse wave is translated into a torsional wave, and vice versa, due to the contact point in the vicinity of the sensors.

One intermediate conclusion is that the slipping causes strong transverse and torsional impulses that are reflected at both ends, but also strongly dampened so that secondary or even further reflections are not notable any more. Another conclusion is that waves of different types are working together, mutually co-determining the action of differential slipping, and the corner rounding of the sawtooth.

### Local perturbation

When inspecting graphs of Figs 6 and 7, all traces for *ß* below 130 mm seem to follow rules of mutual co-operation to form the fundamental Helmholtz cycle, most of which was outlined in the previous sub-section. However, at *ß* = 100 mm torsional motion seems to be reserved, especially when inspecting upstrokes or traces measured at the nut side of the contact point. Transverse and torsional waves might work in the same direction, as has been shown so far, but they might also work against each other.

**Fig 9:**
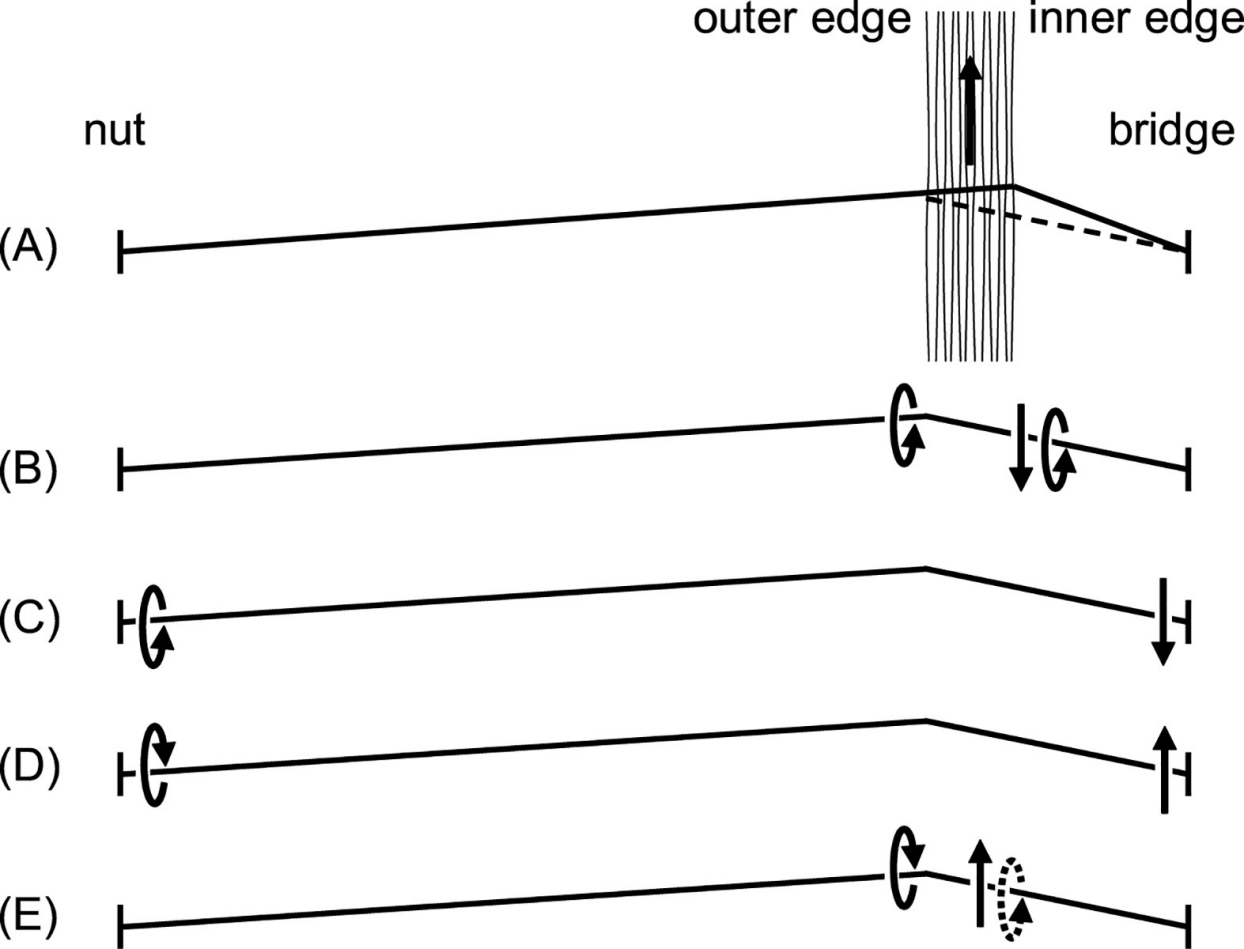
Analysis of local perturbation. Sequence of departure, reflection and arrival of transverse and torsional waves with the potential conflict of directions on arrival at the contact point. For (A) to (E) see text. Conflicting loop times are printed in bold in Table 3.

Following the temporal progress in Fig 9, an intermediate differential slipping will trigger a transverse wave at the inner edge but also a torsional wave, both directed in opposite direction to bowing, Fig 9B. The torsional wave is also present at the outer edge, due to torsional stiffness. The travel time of the torsional wave to the nut is about the same as the travel time of the transverse wave to the bridge, roughly 1.5 ms on this string, see Table 3, Fig 9C. Both waves are reflected and redirected, Fig 9D. On arrival at the contact point, the transverse wave, which is now directed towards the bowing direction, implies a torsional movement in reverse direction due to the contact point in close proximity, indicated by the dotted arrow in Fig 9E. At the same time the torsional wave, which returns from the nut, also arrives at the contact point and is directed towards the bowing direction. Therefore two waves work against each other, effectively lowering the levels. This explains the reserved development of torsional waves at *ß* = 100 mm. It might also explain why some musicians claim, that the sound of bowed strings develops particularly beautifully when played with thick, damping fingers, effectively lowering the impact of the returning torsional wave.

**Fig 10:**
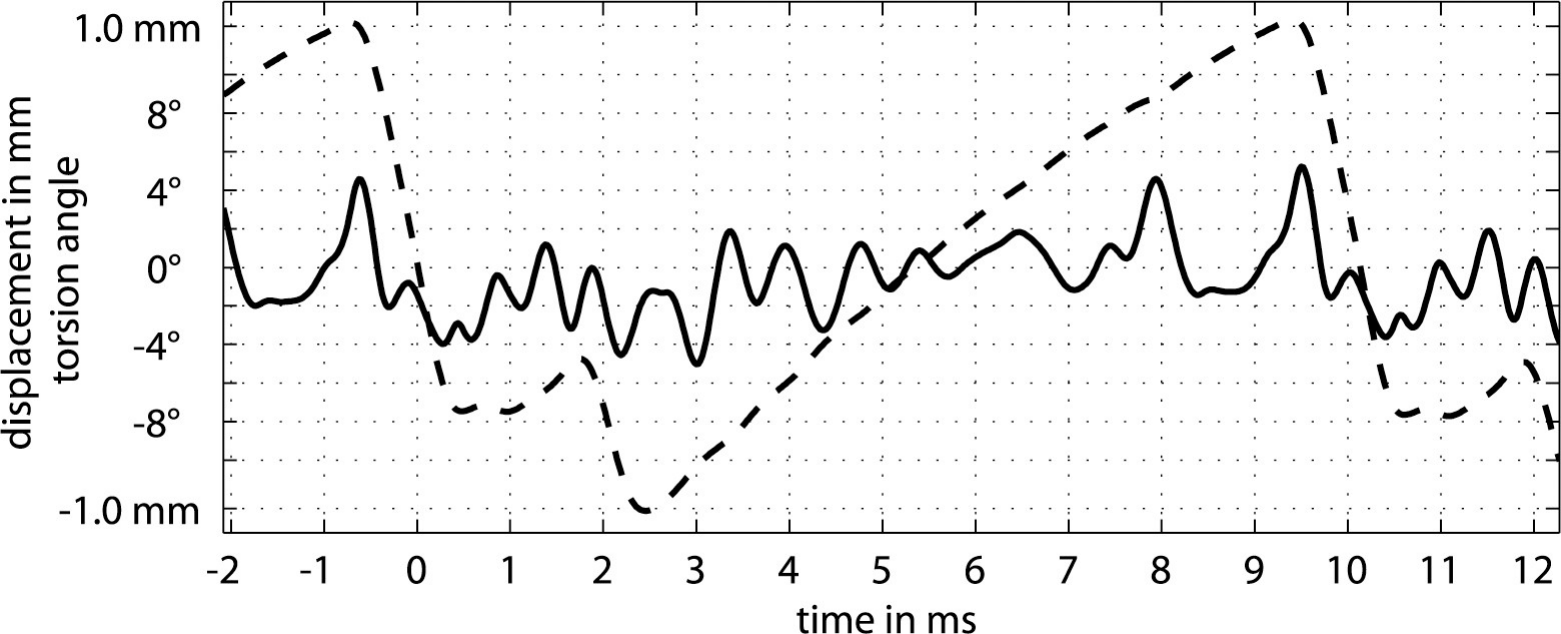
Example of a secondary sawtooth at *ß* = 100 mm. Sample of string displacement (---) and torsion (―) for the case of a secondary sawtooth due to conflicting loop-times at *ß* = 100 mm for the open cello G string.

In fact, during the measurements, the most likely vibration pattern that evolved at *ß* = 100 mm included a second sawtooth, or, in other words, an intermediate recapture at about the mentioned 1.5 ms after the main release (measured 1.3 ms), Fig 10. Obviously the transverse wave returning from the bridge and the torsional wave returning from the nut work together for such intermediate recapturing, since both are heading in the same direction as bowing, Fig 9E. This observation is true for both, down- and upstrokes. It is also true for entire bow strokes including a wide dynamic range in terms of displacement amplitude. At all other bowing positions, on the contrary, such intermediate recapturing was never observed. At *ß* = 100 mm it was also possible to achieve normal Helmholtz motion without intermediate recapturing, and then, again, the vibration pattern was stable along the entire stroke whatever bowing dynamics. Figs 6 and 7 use the cases of normal Helmholtz motion. In conclusion, torsion plays a significant role for the stick-slip-interaction and therefore for the transverse wave and the resulting sound. Viewed on the higher systemic level, at this specific *ß* there are two stable patterns of vibration, a case of bifurcation, which are both surprisingly independent from dynamics, which are stable across the entire bow stroke, and which are obviously directed during onset.

Another divergence from regular patterns, or agreement between the different graphs in Figs 6 and 7 is observable at *ß* = 40 mm when comparing up- and downstrokes measured at the bridge side with those measured at the nut side. While pre-release torsion is generally stronger on the bridge side for a wide range of *ß*, at 40 mm the torsion is stronger on the nut side. The reason for this might be the vicinity of the bridge. While the bow depresses the string, the section towards the bridge is steeper than the section towards the nut, by geometry, see Fig 16 in [16] and Fig 16 in [24]. Therefore, the inner edge of the bow hair effectively experiences more bow pressure than the outer edge. This in return causes the differential slipping to rather happen at the outer edge as the bowing position approaches the bridge. The outer edge is directed towards the nut side and therefore the differential slipping for little *ß* can be identified in the data taken on the nut side, compare Figs 6B and 7B against 6A and 7A. The force conditions and the contact at the inner edge also explains why for little *ß* the torsion at the inner edge just follows the force conditions of the fundamental cycle, see Fig 6A.

In summary, vibrations for bowing in the range below *ß* = 130 mm seem to follow a common pattern of mutual interaction. One cesura is that differential slipping changes from the inner edge to the outer edge when *ß* is small. The other cesura is local perturbation of torsional waves where the transverse to torsional wave speed ratio is *v*_*tra*_ / *v*_*tor*_ = *ß* / (1 - *ß*). This might be the reason why the finger board ends at about 100 mm away from the bridge, which suits the relations found for this steel string.

### Phase relations during release

This sub-section explores which of the transverse or torsional impulses on either side of the contact point is likely to trigger actions of slipping or differential slipping. The phase relations are believed to contribute some arguments.

**Fig 11:**
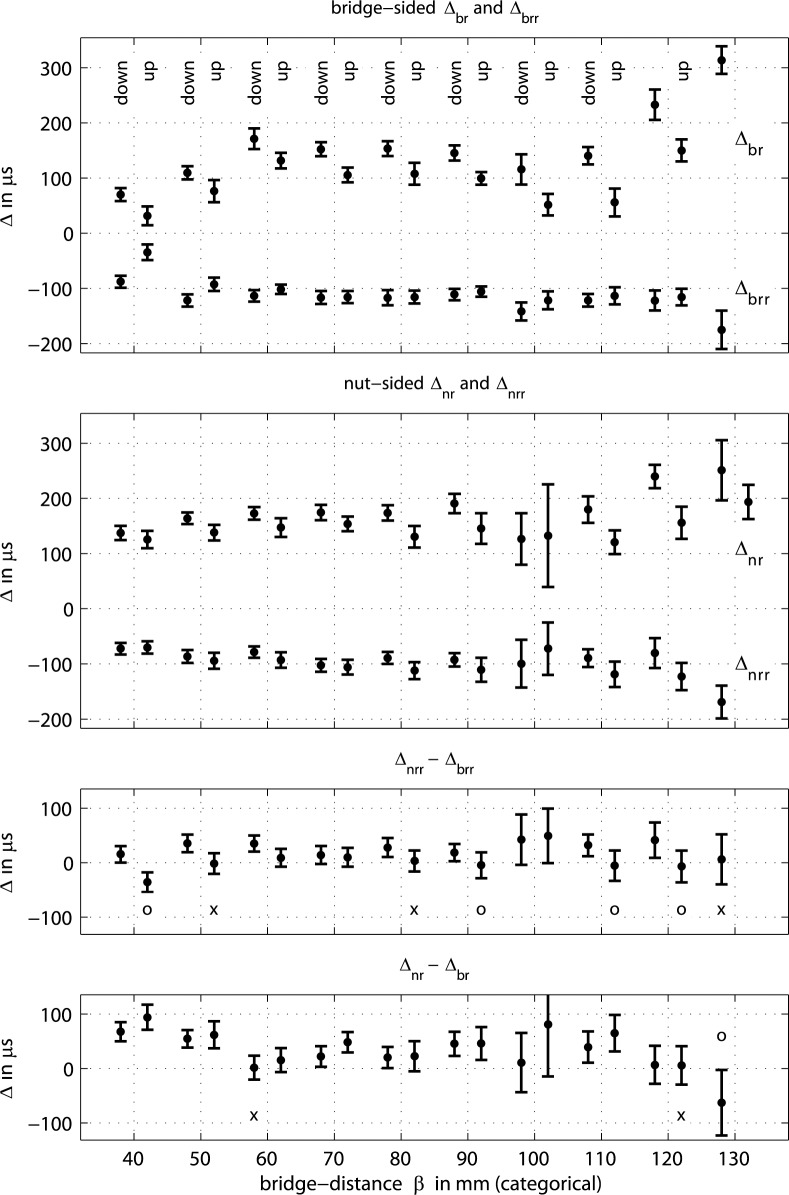
Phase relations between peaks of interest in the course of bow release on an open cello G string. For the individual time differences *Δ* in the upper two graphs see text, peaks of interest are indicated in Fig 4. By comparison of time differences *Δ* in the lower two graphs peaks on the bridge side appear always significantly earlier than peaks on the nut side unless where indicated: (**×**) no significance, (**○**) opposite direction, peaks appear earlier on nut side.

Fig 11 summarizes measured phase relations for the release action. In the top graph, *Δ*_*br*_ denotes the time difference between the turning points of the torsional and the transverse waves, measured at the bridge side during release, *Δ*_*br*_ = *t*(TR)—*t*(DR). Likewise, *Δ*_*brr*_ denotes the time difference between turning point of the torsional wave in reverse direction that precedes the turning point of the transverse wave, *Δ*_*brr*_ = *t*(TRR)—*t*(DR), measured at the bridge side as well. The upper middle graph summarizes the same phase relations, but measured at the nut side, *Δ*_*nr*_ = *t*(TR)—*t*(DR), and *Δ*_*nrr*_ = *t*(TRR)—*t*(DR). The histogram in the bottom left graph of Fig 4 now translates to *Δ*_*br*_ = 20.8 μs · (5.2±0.9) = 108 ± 19 μs for the upstroke at *ß* = 80 mm, for instance. The empirical distribution behind each entry turned out to be normal as a result of Kolmogorov-Smirnov tests.

Every entry represents the mean and the standard deviation of phase relations across 250 fundamental cycles, for each down- and upstroke at each position. Bow force and bow velocity are *not* set to a single predefined value for the 250 cycles. These two parameters are altered during the manual bowing such that a wide dynamic range is covered. The dynamic range, measured in terms of displacement amplitude, covers a factor of minimum two for all strokes. It turns out that these two otherwise important parameters do not matter very much for the measured phase relations, which gets clear when inspecting the standard deviation. Therefore, bow force and velocity are purposely varied to convince the reader that subsequent conclusions do not only hold for a singular instrumentation setup but do hold for a wide range of manual playing. Pitteroff and Woodhouse, on the contrary, claim that the time-keeping mechanisms depend on velocity and bow force, see p. 756 in [15].

While inspecting the two upper graphs of Fig 11, among the many observations the ones relevant for the purpose of this paper are,

- Pre-release reverse torsion: *Δ*_*brr*_ is always roughly 100 μs for *ß* in the range from 50 to 120 mm, and the standard deviation is always less than 18 μs. Therefore, there is a specific phase relation between the torsional waves and the event of release across the playing range, independent from *ß*, bow force and velocity, but probably dependent on the string properties and the tuning. More precisely, the release event is always preceded by a reverse torsion of the string.
- Diversely ruled torsion during slip: *Δ*_*br*_ likewise follows a clear pattern, even though the standard deviation is larger. The torsion turning point TR, which is directed towards the bowing direction and which is always located in the beginning of the slip phase, follows roughly 250 μs after the preceding reverse turning point, TRR. At the same time, TR seems to be ruled by the reflection of TRr at the bridge which becomes clear when comparing the time-lines with those in Table 3 for *ß* = 40 mm and 60 mm, where TR and TRr are close together. The same accordance holds for *ß* = 120 mm, where TR and TRr are further apart. In the range from 60 to 110 mm, TR as a response to TRr seems to be ruled by other effects as well, transverse waves or waves in the bowing stick, which has not yet been further investigated.
- Distinction on the bridge side: the corresponding *Δ*_*nr*_ and *Δ*_*nrr*_ at the nut side are similar to *Δ*_*br*_ and *Δ*_*brr*_ at the bridge side, see the upper center graph of Fig 11. This is not surprising due to the given torsional stiffness of the string, while the distance between the two measurement positions for torsion is only 30 mm. However, there are differences. Torsional waves are less ruled by bridge-sided reflections, as adduced for observation *b)*. The standard deviation is larger at almost all *ß*. This observation might suggest that action or synchronizing events are more likely to happen on the string section at the bridge side while the string section at the nut side follows. Likewise, the more regular temporal structure on the bridge side, Figs 6A and 7A, compared to the nut side, Figs 6B and 7B, suggests the same.
- Local perturbation: the standard deviation for *Δ*_*nr*_ and *Δ*_*nrr*_ is much larger at *ß* = 100 mm than at other bowing positions, some 45 μs for *Δ*_*nrr*_, and some 45 and 90 μs for *Δ*_*nr*_ for down- and upstrokes, respectively. In contrast, the standard deviation for all other observations at *ß* ≤ 90 mm ranges from 10 to only 27 μs. The reason for the less distinct action is believed to relate to the local perturbation, described in the sub-section above.
- Prior action on the bridge side: the lower center graph in Fig 11 represents the difference between *Δ*_*nrr*_ - *Δ*_*brr*_. This difference indicates whether the reverse torsion happens earlier at the bridge side or at the nut side. The standard deviation slightly increases for the calculated difference at each *ß* due to the two given distributions. In almost all cases, the reverse torsion happens earlier at the bridge side by typically 10 to 25 μs. This time difference suits the fact that the travel time of torsional waves from the inner edge of the bow to the bridge sided pickup is 14 μs shorter than the travel time from inner edge to the nut-sided pickup. This earlier action is significant as suggested by the rejected null hypothesis for the distributions of *Δ*_*nrr*_ and *Δ*_*brr*_, at 5% significance. There are only three exceptions where the null hypothesis is not rejected, indicated by (x) in the graph. Four other exceptions attest a faster action at the nut side, indicated by (o) in the graph. These exceptions can be excluded from argumentation because of the proximity to the bridge and to the local perturbation. Apart from these exceptions, the torsion happens significantly earlier at the bridge side as compared to the nut side. In terms of causality, an action is an eligible cause for a subsequent action, but not the other way round. Therefore, the torsional impulses in the vicinity of the release are trusted to be caused on the bridge side. This is in agreement with the observation c). It is also in agreement with the findings of Pitteroff and Woodhouse, p.756 in [15], that the differential slipping happens at the inner edge, and is the motor behind transverse release ripples. The bottom graph in Fig 11 represents *Δ*_*nr*_ - *Δ*_*br*_ indicating where the torsion during the beginning of the slip phase comes first. The prior action on the bridge side is even more apparent here.
- Up-/downstroke divergence: it is apparent that there are differences between up- and downstrokes. The response time between TR and TRR, which can be figured out by *Δ*_*br*_ - *Δ*_*brr*_ in the top graph or by *Δ*_*nr*_ - *Δ*_*nrr*_ in the upper center graph, is always significantly shorter for upstrokes than for downstrokes. The only exception, again, is for the local perturbation at *ß* = 100 mm. This significant difference is similar on the bridge side and the nut side, but, again more distinct on the bridge side. Note that this convincing accordance is given despite manual operation on different days and despite the intermittent instrumentation refitting. The significant difference between up-and downstrokes is not further studied here, however, it is apparent that future stick-slip interaction modelling should incorporate the dynamics of the bow. This opens an interesting perspective for formulating bow dynamics beyond what has already been found by modal analysis [25].

The question now is, which of the waves is dominating anything, and on which side? Inspection of Figs 6 and 7 suggests, that there are no harmonic torsional waves except when bowing at *ß* = 130 mm, or, in general, when bowing in the proximity of *ß* = *l*/5, see the following main section. Rather, there are both, transverse and torsional impulses caused by the release action which are reflected at both ends and which mutually interact.

So far, the first sub-section described the cooperation of reflected transverse waves during slipping in two cases, (i) the reflection from the bridge intensifies the differential slipping, which is synchronized with the fractional *m-HM* cycle, and (ii), the reflection from the nut intensifies the differential slipping which is always preceding the release in the course of the fundamental cycle. From the phase studies in this sub-section it is evident that the torsion resulting from differential slipping is a solid precursor, and that the causing action is on the bridge side.

Before reasoning how the transverse wave that arrives at the outer edge could possibly co-determine the differential slipping happening at the inner edge there is another even more puzzling question: why does the timing of the *m-HM* cycles always coincides with the release action? If it was the Schelleng ripple, the synchronization would come from previous cycles, see Fig 1A, and the question would be answered. However, from the discussion in the first sub-section and from the discussion on phase relations investigated here it is evident that (i) the cooperation of reflected transverse waves and differential slipping is the fundamental action, not the Schelleng ripple, (ii) this action takes place on the bridge side, (iii) the growing ripple comes from the force that increases during the course of the cycle, not supporting the argument of younger versus older Schelleng ripples. These *m-HM* cycles are, if at all, derived from the previous capture action, and they do not necessarily coincide with an upcoming release, see Fig 1A.

There might be a single answer to both questions. The transverse wave which, reflected from the bridge, forms the main cycle, and the transverse wave of the Schelleng ripple have the same direction on arrival at the outer edge. So whatever a reflected transverse wave can do for the main cycle it can also do for intermediate *m-HM* cycles. The argument now is that the *m-HM* cycle gets synchronized with the returning Schelleng ripples. Both, the *m-HM* cycle and the difference between a total loop-time and the fractional loop-time of a Schelleng cycle are of same length anyway and therefore likely to synchronize, see Fig 1A and 1B. There is a strong argument for this synchronization: several observations prove the differential slipping to take place at the inner edge, in agreement with above mentioned publications, and the cycles perfectly merge with upcoming releases and not with preceding captures. In fact, Fig 1A, recalled from mentioned publications, turns out to be misguiding. The observations across all parameters of bowing show that *m-HM* cycles and an upcoming release are synchronized, see Fig 1B. The declining *m-HM* cycle resulting from a recent release-capture action, as suggested, cannot be observed here, see Figs 6, 7 and 8. So the only explanation is a synchronization of the *m-HM* cycles, which are driven on the bridge side, with the Schelleng cycles, which might exist and reverberate on the nut side.

While exploring possible mechanisms of synchronization, the discussion regarding Fig 8 should be recalled. A stronger-than-usual reverse torsional motion at the end of the *m-HM* cycle was explained with the transverse wave which is reflected from the bridge and which is just about to arrive at the contact point. The arriving transverse wave and the given contact point cause torsion into the same direction as does the differential slipping. Having this in mind, one could search for the Schelleng waves on the nut side which should translate to a torsion in bowing direction given the direction of the reflected transverse wave on arrival at the contact point. Such peaks can be observed just before differential slipping, indicated by solid asteristcs in Fig 8, while the timing, which depends on *ß*, suits the fractional loop-time of a Schelleng ripple. An additional analysis on the origin of these peaks shows, that these are also caused on the bridge side: for all cases of *ß* ≤ 120 mm the peak appears significantly earlier on the bridge side as compared to the nut side. The measurements do not allow at present to disclose the mechanism of synchronization, however, such further analysis reconfirms that the motor behind the growing ripples is on the bridge side.

To summarize this sub-section, (i) releases are always preceded by a reverse torsional motion at the contact point, the timing is very distinct, (ii) the reverse torsional motion is induced by differential slipping, because the relevant torsional impulses appear earlier at the bridge side compared to the nut side, (iii) the phase of the *m-HM* cycles driven by differential slipping is not derived from a recent capture, as believed so far, but aligns perfectly with an upcoming release, see Fig 1B, (iv) therefore, the sequence of Schelleng ripples returning at the outer edge is believed to synchronize the *m-HM* cycles while the action takes place at the inner edge.

### Phase relations during capture

**Fig 12:**
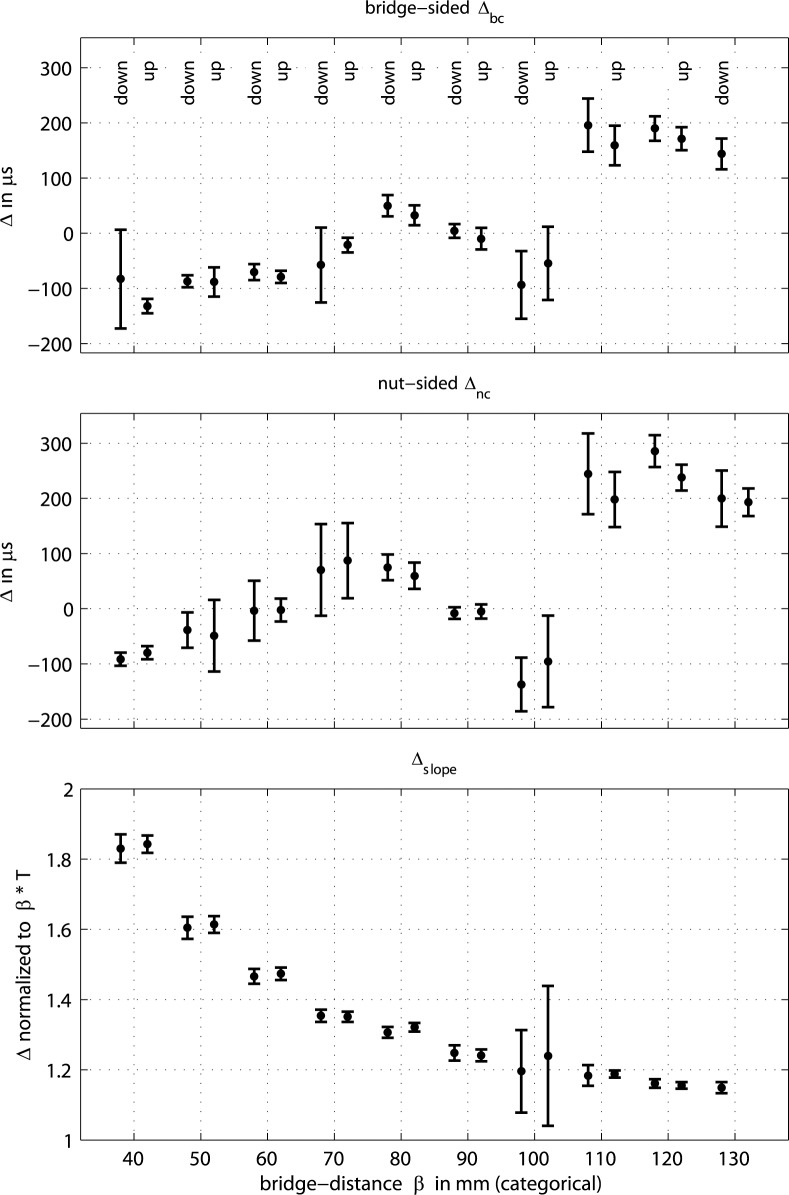
Phase relations between peaks of interest in the course of bow capture on an open cello G string. For the individual time differences *Δ* in the upper two graphs see text, peaks of interest are indicated in Fig 4. The bottom graph shows the slope time *Δ*_*slope*_ = *t*(DC)—*t*(DR), normalized to the relevant fraction of the fundamental period, *ß · T*.

Fig 12 summarizes phase relations during capture. Again, *Δ*_*bc*_ denotes the time difference between turning point of the torsional wave that precedes the turning point of the transverse wave during capture, *Δ*_*bc*_ = *t*(TC)—*t*(DC), when measured at the bridge side, and *Δ*_*nc*_ = *t*(TC)—*t*(DC), when measured a the nut side. Likewise, the histogram in the bottom left graph of Fig 4 translates from discrete samples to time, *Δ*_*bc*_ = 20.8 μs · (1.5±0.9) = 33±18 μs for the upstroke at *ß* = 80 mm. The statistics cover the same *n* = 250 cycles of each stroke as in the analysis of phase relations during release.

Contrary to the release action, the capture action reveals clearly less of a stringent phase relation between displacement and torsion. Here, the turning point of torsions in bowing direction is considered for a good reason: it is assumed that such torsion could possibly support the mechanism of capturing, simply because the string, when at a low slip velocity relative to the bow, is more likely to find grip when the torsion is directed towards the bowing direction. On the contrary, a reverse torsion effectively increases the slip velocity, and gripping is less likely. Having this in mind, the analyzed phase relations do not support the idea that capturing is co-determined by torsion. While for the release action, the differential slipping happens in a time-window of 110±10 μs prior to release across almost the entire playing range, the span for the capture stretches from -150 to 300 μs. Only at *ß* = 80 and 90 mm the torsion happens to coincide with the displacement turning point, *Δ*_*nc*_ ≈ 0, effectively supporting the action. The very small standard deviation supports the argument, when we assume that well-synchronized events are more distinct. The torsion is rather a response to the release action, as can be observed by checking the nut-sided loop-time for torsion in Figs 6 and 7. This nut-sided loop-time decreases with *ß* while the displacement slope increases with *ß*. At *ß* = 80 and 90 mm the slope duration and loop-time match.

The bottom graph of Fig 12 shows the slope duration measured from the turning point during release to the turning point during capture, *Δ*_*slope*_ = *t*(DC)—*t*(DR), normalized to *ß · T*, with *T* representing the fundamental period. One would expect unity for the normalized *Δ*_*slope*_ across all *ß*, assuming an ideal sawtooth and a single-point contact. However, the normalized *Δ*_*slope*_ only approaches unity for larger *ß*. This might be an important note for future modelling.

Another observation worth mentioning is the large standard deviation at *ß* = 100 mm. The jitter of the fundamental cycle is generally believed to be low [9]. However, in cases where *v*_*tra*_ / *v*_*tor*_ = *ß* / (1 - *ß*) jitter may be leveraged. The standard deviation of the relative slope duration is a factor of almost 10 larger at this playing position compared to all other playing positions. This is where local perturbation hampers the mutual synchronisation otherwise found at the other bowing positions.

In summary, torsion seems to play an inferior role during the capture mechanism. Torsion is determined during the release action and reflected and returning waves just happen to have this or that phase relative to displacement. If torsion coincides with capturing, the action is temporarily more distinct. Slipping slopes are wider than expected, especially at low *ß*. Local perturbation enlarges jitter.

### Subharmonics

Subharmonics are reported to be audible and are believed to be caused by suitable sequences of wave reflections at the contact point, in times of sticking, and transmissions across the contact point, in times of slipping, see Fig 1C, following Fig 11 in [9]. McIntyre et al. also report sub-harmonics measured magnetically at the contact point, see Fig 7 in [9], at least for a few sub-harmonic cycles. The visual inspection of Figs 6 and 7, however, rather suggests that there are strong impulses caused by the release, which are then reflected only once or twice. It is not recognizable that any *m-HM* transverse or any torsional wave would prolong its existence beyond the present or even one further fundamental cycle. From the analysis of loop-times that can be identified, there are no such waves that would keep on going over several fundamental cycles. Both, transversal and torsional waves decline strongly, and an upcoming release will always overrule past and fading waves. This observation is reasonable, because the bow hair is in fact damping the string, whether slicking or slipping.

Apart from observation, there is also a rational argument against the concept of subharmonics presented in Fig 1C. Assuming that waves could indeed follow such an imagined travelling path along the string over the period of four or five fundamental cycles, why should they be generated only every four or five cycles? They are likely to be generated every cycle, and the final result after superposition would be equality for all cycles, or traces, without notable differences every four or five cycles.

For the purpose of identifying potentially existing subharmonics in the given data set, waves are statistically examined. Only torsional waves are examined, because the traces of torsion also capture transverse waves, as has been shown in the previous main section. Minor ripples in the trace of transverse motion are even more apparent in the traces of torsion. For beforehand visual examination, sections of 100 fundamental cycles serve as the fundamental set for each up- or downstroke, and for each *ß*. The sampled raw data of a complete fundamental period is treated as one entity with time reference to the zero-crossing of displacement during slipping. In search of a 1/*n* subharmonic, averages over every *n*th cycles are calculated. Precisely, *n* sets from the numbered *i* = 1 … 100 cycles are taken such that they obey *i* mod *n* = *j* to belong to the same set number *j*, with *j* = 0 … *n* -1. Entities belonging to the same set are then averaged. Visual examination of the averaged entities suggests that there are only minor differences, but if there are any, these differences are present across the entire cycle and are always most apparent during peaks, i.e. at TR and TCR. Therefore, for statistical examination, non-averaged samples of TR are used, one from each cycle, belonging to respective sets. The null hypothesis assumes that any individual set would not significantly differ from any other set to identify subcycles of order *n*. A less extensive test paradigm was used while only checking each set against the aggregate of all remaining *n*-1 sets.

On basis of the Wilcoxon ranksum test, the null hypothesis was not at all rejected for *n* = 4. It was rejected in few cases out of the 26 up- and downstrokes at the various *ß* for *n* = 5, and it was often rejected for *n* = 6, see Table 4. The two-sample Kolmogorov-Smirnov test, as an alternative method, delivers the same results in almost all cases.

**Table 4:** Observations of rejected null-hypotheses indicating existing 1/*n* subharmonics in a total of 26 up- and downstrokes at varying positions in the playing range based on 100 fundamental cycles each.

*n* =		4	5	6	7
bridge-sided	*α* = 0.05	0	2	12	0
	*α* = 0.1	0	1	5	3
nut-sided	*α* = 0.05	0	3	0	1
	*α* = 0.1	0	1	0	2

As a result, subharmonics, if at all, can be observed for *n* = 6. While McIntyre et al. reported audible subharmonics preferably for rather large *ß* (1/3 and 1/4), the observations here are randomly distributed across all *ß*. Strikingly, *n* = 6 closely relates to the ratio of propagation velocities, *v*_*tor*_ / *v*_*tra*_ = 738 ms^-1^/ 133 ms^-1^ ≈ 5.55. Following the argumentation of local perturbation in the respective sub-section, and given the measured loop-times, the transverse and torsional waves are likely to mutually interact every six cycles.

This study does not prove or give an explanation to how subharmonics are likely to happen. However, this study identifies an alternative perspective.

### Data outside the regular playing range and flageolet tones

In Figs 6 and 7, standing torsional waves are identifiable for *ß* = 130 mm and *ß* = 140 mm. Here, peaks of torsional motion seem to be equally spaced, five of them for each fundamental cycle. The five-fold fundamental frequency relates to the bowing position. The perfect knot location for a flageolet tone at 1/5 of *l* is at 0.68/5 m = 136 mm, just in between the two mentioned positions. There exists a wider discussion on bowing in the proximity of *l*/*n* locations, or for S-motion in general, including the dedicated study by Lawergren in [26]. Here, only a few observations should be mentioned regarding of the role of torsion and the potential difference between steel and gut strings, not addressed by Lawergren.

For the given open string, a standing torsional wave would oscillate at 543 Hz, see Table 1. However, when bowing in the proximity of *ß* = *l*/5, a flageolet tone at 5 x 98 Hz = 490 Hz develops. The transverse wave at 490 Hz dominates the torsional wave at 543 Hz, or, the torsional wave synchronizes and its frequency shifts down to that of the transverse wave. At this point it is good to remember, that torsional waves are dampened much more than transverse waves, and that the torsional waves are therefore more likely to be enslaved by the transverse waves rather than the other way around. Likewise, the 5th harmonic, which can be considered as just another transverse wave, is enslaved by the fundamental, while the bowing position is off the perfect knot location. Such synchronization mechanisms including frequency shifting are reported for organs in [27], [28], and [29], and are generally discussed in a systematic approach by Bader [30].

At *ß* = 150 mm, the bowing position is too far from the mentioned spot, and the 5th harmonic does not develop any longer. If any, rather the 4th harmonic should develop while gradually approaching *ß* = *l*/4. The torsional motion here seems to be less dependent from the transverse motion. There are seven major torsional peaks, and, notably, sections of suppressed peaks, or, apparent phase-shifts, Figs 6 and 7. The torsional motion on its own would be harmonic, but here, it is ruled by transverse waves in a non-harmonic but deterministic way.

Searching for further spots of synchronization reveals that in close proximity, again, of *l*/3 and of *l*/6 standing torsional waves develop. At *l*/6, these are somewhat more difficult to be achieved by bowing, but they can be purposefully produced. At *l*/3 torsional waves are easily produced. Surprisingly, the standing torsional waves oscillate at six times the fundamental frequency, in both cases. At *l*/3, obviously, the torsional wave synchronizes with every second beat of the third overtone. The string’s torsional vibration, nominal at 5.55 times the fundamental, can be synchronized to five or six times the fundamental frequency, but not necessarily to three times the fundamental frequency.

Searching for the spots of such mutual synchronization on gut strings reveals a surprise. On a Damian Dlugoleck1 cello G gut string, when bowing in close proximity of an *l*/*n* position, always the *n*th overtone develops, *n* = 2 ..5. The only difference while bowing is, that the bowing position is further away from the nominal knot position compared to the experiment with the steel string. The more important and surprising difference is the result that, obviously, the torsional vibration on a gut string is more willing to follow existing transverse motions, reaching for frequencies further away from nominal values. The higher internal damping of gut strings as compared to that of steel strings corresponds well with the observation of a wider span of synchronization.

While torsional waves synchronize to transverse waves, on the steel string they are more likely to stick close to their nominal frequency while on the gut string they are more likely to abandon their nominal frequency.

To summarize, there are no standing torsional waves apart from cases where transverse waves, and possibly related overtones, mutually synchronize with the torsional motion. Transverse waves dominate torsional waves, obviously even more on gut strings than on steel strings.

## Recapitulation and discussion

### Recapitulation of observations

Following observations are evident and should be reproducible without difficulties:

There are no standing torsional waves, apart from when torsional motion nicely synchronizes with transverse motion, e.g. when bowing in the proximity of *l*/*n*.

The release is always preceded by a strong reverse torsional motion. The span between these two events is distinct, the standard deviation is always less than 18 μs across the entire playing range for the open cello G string. This phase relation does not alter with *ß*.

The release is also synchronized with the final of a sequence of *m-HM* cycles. These cycles are generated on the bridge side as can be proved by the phase relations of bridge-sided versus nut-sided torsions, across the playing range. These cycles are therefore likely to be caused by differential slipping.

Across the playing range, *m-HM* cycles are always accompanied synchronously by a pair of a reverse and a forward torsional motion. This also relates to differential slipping, since the partial release of the string will not only cause fractional backward slipping but also fractional reverse torsion, since the applied and then released forces work for both.

Transverse and torsional motions may adversely affect each other. In the case of matching travelling times for torsion on the nut side and transverse waves on the bridge side, the standard deviation of the duration of the release slope increases by a factor of almost ten. In other cases Helmholtz motion might even be entirely obstructed, e.g. when bowing at *l*/*n*.

There are differences between upstrokes and downstrokes with quite some regularity across the playing range, expressed by phase relations of travelling waves. These differences have not been studied further, but it is apparent that future stick-slip modelling should address this.

The following less evident but plausible observations were found:

Subharmonics, or deviations of one fundamental cycle versus adjacent cycles in a repeated fashion every *n* cycles are significantly present, when *n* matches about the relation of *v*_*tor*_ /*v*_*tra*_. The argument is strengthened by the other observation that flageolet tones of order *n* do not necessarily develop when bowing in the proximity of *l*/*n*. On the steel string the stick-slip interaction caused a flageolet tone of order *n* that again matched about the relation of *v*_*tor*_ /*v*_*tra*_, even though bowing happened at 2*l*/*n*.

### Observations related to earlier concepts

The findings should now be related to earlier concepts of bowed string interaction. First of all, this study delivers many different kinds of observations on synchronization. String motions are willing to surrender to each other, to give up nominal frequencies, and so on. Second, there are no observations of waves that would extend their vibrational pattern beyond an upcoming release-capture action. The release action is the dominant one, furnishing transverse and torsional waves with energy, and there are typically only one or two reflections before a wave diminishes within the given fundamental cycle.

These observations are only so clear because this study captures motion in the vicinity of the bowing point. On the contrary, the commonly practiced measurement of bridge force leaves room for interpretations. These always require assumptions on how waves are truly travelling, including assumptions on which wave, or which part of a wave, is reflected by or passing through the contact point. Here, signals are directly captured. Another leveraging is the measurement of torsion. String torsions are not likely to be observable in bridge force measurements. The traces of torsion also hold information on transverse motion, in the same signal, since the two types of motion are coupled in the vicinity of the contact point. In conclusion, there are less speculations and there is enough resolution to reason phase relations and causes.

One of the main findings is that the concept of Schelleng ripple is under question. The growth of these ripples was explained so far with the older vs. younger argument resulting from the reverse-order-of-appearance at the contact point, Fig 1A. However, when comparing the bridge side with the nut side, the timing analysis proves that the action is happening at the bridge side. The growth is plausible, since differential slipping gains strength during the course of the fundamental cycle as the applied forces increase with displacement. The temporal patterns show that the periodical slipping is in line with the upcoming release and not with the past capture, Fig 1B. Observations and arguments calls the concept of Schelleng ripples into question.

The concept of subharmonics likewise trusts that waves can exist over a longer sequence of fundamental cycles. The observation here was rather that every release has such a strong impact that all waves of all types are re-written. Statistical analysis also suggests that the few observed deviations of every *n*th cycles from other, adjacent cycles is contributed to the relation of wave speed. The existing concept of subharmonics implies dependence from *ß* which cannot be confirmed by the statistical analysis. This study therefore introduces another perspective on subharmonics.

Different slipping can be fully confirmed since all descriptions related to differential slipping appear to be consistent with present observations. There are indeed strong torsional vibrations generated during the course of a fundamental cycle which can only come from the action of differential slipping. And indeed the action takes place at the inner edge of the bow.

However, the concept of forward slipping at the outer edge, in the context of differential slipping, cannot be shared. The general assumption for this model is a single residual point or area of sticking within the limited width of bow hairs. There should be no problem with this assumption, but it leads to a different conclusion for the given mechanisms. The backward slipping at the inner edge comes along with a reverse torsional motion. This reverse torsional motion is also present at the outer edge due to the given torsional stiffness. At the same time, the given bending stiffness forces opposite transverse motion across the contact point: backward motion on one side forces forward motion on the other side. The resulting signs now suggest a forward rolling at the outer edge rather than a forward slipping, because at the inner edge backward transverse motion and reverse torsion both promote slipping, while forward motion in combination with reverse torsion at the outer edge results in rolling.

Finally, there is the question on the impact of torsion on vibrational modes and perception, as compared to the impact of varying friction models. The list of observations for torsion is,

- Determination of temporal precision: torsional vibrations proved to sharpen ongoing transverse waves in terms of temporal matters. This was observed for specific playing positions when torsional and transverse vibrations cooperate, see standard deviations of phase relations between torsion and transverse waves, and the standard deviation of slope times of the transverse wave. Torsion is indeed inferior to the transverse vibrations in terms of impedance but its contribution is significant. Temporal precision, or the obviously harmonizing cooperation might also leverage stability or the ease of playing.
- Perturbation of transverse motion: the mechanism of local perturbation has been outlined, when torsional and transverse impulses act in opposite direction while returning to the contact point. The related specific *ß* is determined by the travel times. This adverse action results in vibrational modes that in general come along with lower amplitudes for the torsion, the adverse action might also affect the stability bowing or the ease of playing. The effect on transverse motion is evident: there are two different stable vibrational patterns at the same *ß*, the regular Helmholtz sawtooth and a double sawtooth, both independent from bowing dynamics.
- Overtones on gut versus steel strings: it was observed for the steel string that the order *n* of flageolet tones that developed matched about the relation of *v*_*tor*_ /*v*_*tra*_, independent from whether the playing position was close to *l/n* or not. This was not the case for the respective gut string where the *n*th flageolet tone strictly developed when playing close to *l/n*. The importance of overtones of gut strings for baroque music is known and can possibly be explained by the damping of adverse torsional modes.
- Pre-release reverse torsion: reverse torsion is a reliable precursor of the main release, and its timing does not depend on *ß* when bowing in the normal playing range. While the mechanism behind the mutual synchronization is not understood yet, the existence of the mechanism is evident.
- Corner-rounding: the often discussed corner rounding is contributed to torsion, at least to some extent. This can be seen in Fig 4, every ripple in the transverse motion is strictly related to a torsional motion. At the Helmholtz corners the torsional vibration peaks. Corner rounding has been discussed as relevant for timbre.

While observations *c)* and *e)* clearly translate to perceptible changes of the tone or its timbre, it is less clear for observations *a)* and *b)*, *or d)*. The reader might wish to study manual bowing at opportune versus less opportune positions (*ß* = 80 or 90 mm versus 100 or 110 mm) to search for differences. The author observed faster onsets for the opportune positions but only when playing on the monochord, and less so when playing on a real cello.

Mansour et al. [6] claim that the choice of the friction model is by far the most sensitive parameter to bow force limits, pitch flattening and relative spectral centroid. No other modeling parameter, including the option to employ torsion or not, would change the simulation outcome as much as would the friction model. Given the above mentioned observations and arguments it seems interesting of whether matters of friction, or the rosin, or whether matters of torsion are more likely to shift vibrational modes. This question led to a series of some additional experiments of manual bowing. A setup was designed to deconstruct vibrational modes at a termination point for better understanding. The finger termination was replaced by looped wire with the choice of a single-point contact or a twin-point contact at opposite-sides of the string. The twin-point contact terminates torsion, the single point termination does not. Both options were used with more or less force at the contact point, and in accordance with or perpendicular to bowing direction, at locations close to *l*/4. The bowing response is the fundamental tone or the 4th flageolet tone most of the times, or both, with good portions of noise. The bow force limits vary a lot while developping either tone under the alternative termination options. There is little chance of systemization, but the impression is that varying the termination/reflection of torsional waves has a stronger impact on sound than varying rosin. There is no instrumental evidence yet, but the author is not convinced that matters of friction are by far more relevant to describing stick-slip interaction than are the intrinsic string properties, including torsion. Readers are encouraged to examin the experiment themselfes and researchers are encouraged to empirically validate assumptions of modelling and simulation results.

## Future modelling

Future numerical simulation might address the challenge to model the coupling between the different vibrational modes, and the transformation between them at the points of contact and termination. This might help to understand how the mechanisms of differential slipping truly work and how this gets synchronized with the main cycle. A lot of the corner rounding seems to be contributed by torsion, which should become clearer when simulation covers coupling and transformation between vibrational modes. Differential slipping is a key to the caused vibrational modes and necessary to adequately describe the *m-HM* cycles. A two-point contact model should be enough to represent the actions on the bowed string. Differences between up- and downstrokes are apparent. It might be promising to explore alternative concepts of modelling, for instance formulating impulse patterns in coupled systems rather than forces in a geometrical context.

## Conclusions

Torsional vibrations are measured on both sides of the contact point for varying bowing positions on an open cello G string, and are related to the transverse motion of regular Helmholtz cycles. The structural analysis of coexisting vibrations suggests that these are mutually coupled, ready for transformation or submission while arriving at terminations or returning at the contact point. It also suggests that the release action provides the main energy impulse for vibrations of both types which will then decline fast, i.e. the transverse wave on the bridge side and the torsional waves on both sides. Secondary energy impulses come from differential slipping which, on the contrary, are rather likely to grow due to increasing forces towards the end of the sticking phase.

The concept of differential slipping can be fully confirmed apart from the detail that there is probably no such forward slipping at the outer edge as suggested earlier. The concept of Schelleng ripples cannot be confirmed. The evidence comes from measured phase relations, suggesting that the initiative for the fractional Helmholtz cycle, which is determined by the transverse travel time and distance between contact point and bridge, is clearly coming from actions at the inner edge, on the bridge side, while Schelleng ripples are nut-sided. These fractional Helmholtz cycles are driven by differential slipping and are synchronized with the upcoming release and not with the previous release as believed earlier. This is true for bowing positions across the entire normal playing range and independent from playing dynamics. The existing concept of subharmonics cannot be confirmed likewise. Subharmonics, if at all, can be found at integer ratios close to the ratio of propagation velocities *v*_*tor*_/*v*_*tra*_, independent from *ß*.

Finally, torsional vibrations are relevant to adequate modelling and to perception. While the course of recent publications concludes on the friction model to be the main key for adequate modelling of stick-slip interaction this study concludes that there are many observations that can only be adequately explained with torsion, whatever rosin is used on a bow. Among these are a few which should be directly perceptible: (i) the jitter of the main cycle is determined by torsion, (ii) overtones on the steel string are rather determined by *v*_*tor*_/*v*_*tra*_ while on the gut string they are rather determined by the bowing position, and (iii) for bowing positions which obey *v*_*tra*_ / *v*_*tor*_ = *ß* / (1 - *ß*) both a regular Helmholtz motion but also cycles with two sawtooths can be produced in a very stable fashion, both well within the range of related bow force limits. Another observation is of rather physical nature. Each release action is preceded by a reverse torsion (*σ* = 18 μs) independent from bowing position and from playing dynamics. Torsion therefore clearly co-determines stick-slip interaction and should play a key role in modelling.
